# Genetic suppressor screen identifies Tgp1 (glycerophosphocholine transporter), Kcs1 (IP_6_ kinase), and Plc1 (phospholipase C) as determinants of inositol pyrophosphate toxicosis in fission yeast

**DOI:** 10.1128/mbio.03062-23

**Published:** 2023-12-22

**Authors:** Lauren Bednor, Ana M. Sanchez, Angad Garg, Stewart Shuman, Beate Schwer

**Affiliations:** 1 Department of Microbiology and Immunology, Weill Cornell Medical College, New York, USA; 2 Molecular Biology Program, Sloan-Kettering Institute, New York, USA; 3 Weill Cornell Graduate School of Medical Sciences, New York, USA; 4 Gerstner Sloan Kettering Graduate School of Biomedical Sciences, New York, USA; Harvard Medical School, Boston, Massachusetts, USA

**Keywords:** inositol pyrophosphate, fission yeast, glycerophosphocholine transporter, phosphate homeostasis

## Abstract

The inositol pyrophosphate signaling molecule 1,5-IP_8_ is an agonist of RNA 3*'*-processing and transcription termination in fission yeast that regulates the expression of phosphate acquisition genes *pho1*, *pho84*, and *tgp1*. IP_8_ is synthesized from 5-IP_7_ by the Asp1 N-terminal kinase domain and catabolized by the Asp1 C-terminal pyrophosphatase domain. *asp1-STF* mutations that delete or inactivate the Asp1 pyrophosphatase domain elicit growth defects in yeast extract with supplements (YES) medium ranging from severe sickness to lethality. We now find that the toxicity of *asp1-STF* mutants is caused by a titratable constituent of yeast extract. Via a genetic screen for spontaneous suppressors, we identified a null mutation of glycerophosphodiester transporter *tgp1* that abolishes *asp1-STF* toxicity in YES medium. This result, and the fact that *tgp1* mRNA expression is increased by >40-fold in *asp1-STF* cells, prompted discovery that: (i) glycerophosphocholine (GPC) recapitulates the toxicity of yeast extract to *asp1-STF* cells in a Tgp1-dependent manner, and (ii) induced overexpression of *tgp1* in *asp1*^+^ cells also elicits toxicity dependent on GPC. *asp1-STF* suppressor screens yielded a suite of single missense mutations in the essential IP_6_ kinase Kcs1 that generates 5-IP_7_, the immediate precursor to IP_8_. Transcription profiling of the *kcs1* mutants in an *asp1*^+^ background revealed the downregulation of the same phosphate acquisition genes that were upregulated in *asp1-STF* cells. The suppressor screen also returned single missense mutations in Plc1, the fission yeast phospholipase C enzyme that generates IP_3_, an upstream precursor for the synthesis of inositol pyrophosphates.

**IMPORTANCE:**

The inositol pyrophosphate metabolite 1,5-IP_8_ governs repression of fission yeast phosphate homeostasis genes *pho1*, *pho84*, and *tgp1* by lncRNA-mediated transcriptional interference. Asp1 pyrophosphatase mutations that increase IP_8_ levels elicit precocious lncRNA termination, leading to derepression of the *PHO* genes. Deletions of the Asp1 pyrophosphatase domain result in growth impairment or lethality via IP_8_ agonism of transcription termination. It was assumed that IP_8_ toxicity ensues from dysregulation of essential genes. In this study, a suppressor screen revealed that IP_8_ toxicosis of Asp1 pyrophosphatase mutants is caused by: (i) a >40-fold increase in the expression of the inessential *tgp1* gene encoding a glycerophosphodiester transporter and (ii) the presence of glycerophosphocholine in the growth medium. The suppressor screen yielded missense mutations in two upstream enzymes of inositol polyphosphate metabolism: the phospholipase C enzyme Plc1 that generates IP_3_ and the essential Kcs1 kinase that converts IP_6_ to 5-IP_7_, the immediate precursor of IP_8_.

## INTRODUCTION

**Fig 1:**
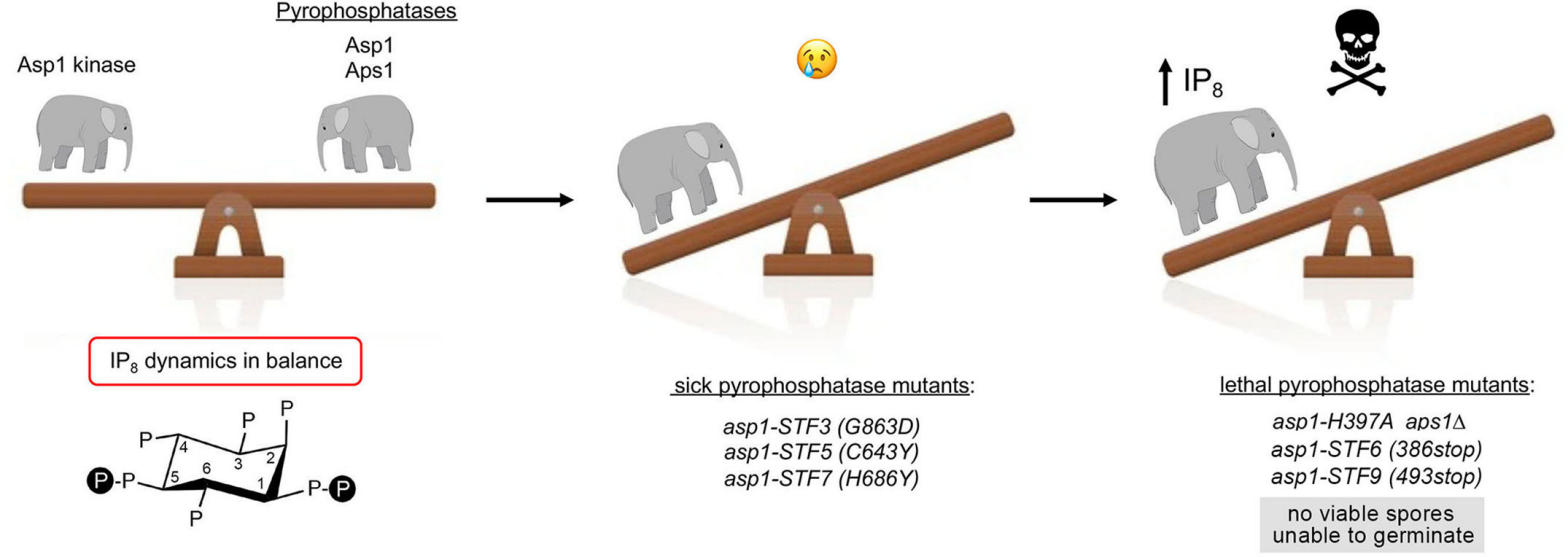
Pyrophosphatase mutations perturb IP_8_ dynamics and growth of fission yeast. Inositol pyrophosphatase mutations lead to increased intracellular IP_8_ (chemical structure shown in left panel) and elicit growth defects in YES medium ranging from severe sickness (middle panel) to lethality (right panel).

The inositol pyrophosphate metabolite 1,5-IP_8_ (Fig. 1) is generated from phytic acid (IP_6_) by the sequential action of two kinases: Kcs1, which converts IP_6_ to 5-IP_7_, and Asp1, which converts 5-IP_7_ to 1,5-IP_8_. In the fission yeast *Schizosaccharomyces pombe*, inositol pyrophosphate levels are dictated by a balance between the two kinases and the inositol pyrophosphatase enzymes Asp1 and Aps1 that remove the β-phosphate groups (Fig. 1). Asp1 is a bifunctional enzyme composed of an N-terminal kinase domain, of the ATP grasp family, that synthesizes IP_8_ and a C-terminal pyrophosphatase domain, of the histidine acid phosphatase family, that converts IP_8_ back to 5-IP_7_ (1–4). Aps1 is a Nudix-family inositol pyrophosphatase (5). A *kcs1*∆ mutation is annotated as lethal in *Schizosaccharomyces pombe* (www.pombase.org/gene/SPCC970.08), signifying that IP_7_ plays an essential role during vegetative growth. By contrast, a fission yeast *asp1*∆ null mutant, which has no detectable IP_8_ (1), is viable.

IP_8_ is a key governor of fission yeast phosphate homeostasis (6), a transcriptional program in which the expression of three genes comprising a *PHO* regulon*—pho1* (cell surface acid phosphatase), *pho84* (inorganic phosphate transmembrane transporter), and *tgp1* (glycerophosphodiester transporter)—is repressed under phosphate-replete conditions by RNA polymerase II (Pol2) transcription of 5*'* flanking lncRNAs *prt*, *prt2*, and *nc-tgp1*, respectively (7). The *PHO* regulon is derepressed in phosphate-replete cells by mutations of the Asp1 pyrophosphatase module that lead to precocious lncRNA 3*'*-processing/termination in response to poly(A) signals upstream of the *PHO* mRNA promoters (6). For example, increasing IP_8_ in pyrophosphatase-defective *asp1-H397A* cells (1) derepresses the *PHO* regulon and prompts precocious termination of *prt* lncRNA synthesis, in a manner dependent on the cleavage and polyadenylation factor complex (CPF), transcription termination factor Rhn1, and the Thr4 phospho-site of the C-terminal domain (CTD) of the Rpb1 subunit of Pol2 (6). Simultaneous inactivation of the Asp1 and Aps1 inositol pyrophosphate pyrophosphatases is synthetically lethal (Fig. 1), i.e., we were unable to recover viable *asp1-H397A aps1*∆ haploid progeny after crossing the two single mutants, signifying that too much IP_8_ is toxic. The lethality of *asp1-H397A aps1*∆ was suppressed by crossing to loss-of-function mutants of CPF subunits (Ppn1, Swd22, Ssu72, Ctf1) and by crossing to *rpb1-CTD-T4A* (6).

Alanine substitution of all CTD Thr4 positions prevents installation of the Thr-PO_4_ mark and, by itself, results in *PHO* hyper-repression (8, 9). A forward genetic screen for mutations that derepress Pho1 acid phosphatase expression in *CTD-T4A* cells (10) yielded 18 independent *STF* (suppressor of threonine four) isolates, every one of which had a mutation in the C-terminal pyrophosphatase domain of Asp1. Crossing the *STF* isolates to wild-type (WT) highlighted a spectrum of growth defects associated with Asp1 pyrophosphatase mutations. Three *asp1-STF* alleles*—STF3* (*G863D*), *STF5* (*C643Y*), and *STF7* (*H686Y*)—caused severe growth defects (“sickness”) on yeast extract with supplements (YES) medium in a wild-type CTD background (Fig. 1). The *STF6* and *STF9* mutations*—asp1-386*(*Stop*) and *asp1-493*(*Stop*), respectively—were lethal in a wild-type CTD background, as judged by the inability to recover viable haploid *STF6* and *STF9* single-mutant progeny in the cross. However, *STF6* and *STF9* were viable when combined during crossing with deletion or loss-of-function mutations in CPF subunits and Rhn1 (10). Taken together, these findings implicated Asp1 pyrophosphatase in constraining IP_8_ synthesis by Asp1 kinase, without which IP_8_ can accumulate to toxic levels that elicit precocious termination of one or more essential fission yeast genes.

To clarify the mechanism of IP_8_ toxicosis and the signaling pathway(s) that connects IP_8_ elevation to Pol2 termination, we have conducted genetic screens for spontaneous mutations that suppressed the “sick” growth of *asp1* pyrophosphatase mutants *STF3* (*G863D*), *STF5* (*C643Y*), and *STF7* (*H686Y*) on YES medium. Via this *SST* (suppressor of suppressor of Thr4) screen, we identified a novel hypomorphic mutation of the essential CPF subunit Cft1 (11). The *SST* screen also yielded loss-of-function mutations in Gde1 and Spx1 (11), both of which have an inositol pyrophosphate-binding SPX domain (12, 13).

Here, we report that the severe growth defects of the *asp1-STF* mutants in YES medium are caused by a titratable constituent in yeast extract. By expanding the scope of the *SST* screen, we identified missense mutations of essential inositol polyphosphate synthetic enzymes Plc1 (phospholipase C) and Kcs1 (IP_6_ kinase) and a null mutation of Tgp1 (a putative glycerophosphodiester transporter) that circumvented the toxic effects of *asp1-STF* alleles in YES medium. RNA-seq revealed that *tgp1* mRNA expression is increased by 43- to 47-fold in *STF6* and *STF9* cells, respectively. These findings led us to discover that glycerophosphocholine (GPC) recapitulates the toxicity of yeast extract to *asp1-STF* cells in a Tgp1-dependent manner.

## RESULTS

### *asp1-STF6* and *STF9* strains are defective for outgrowth after spore germination

**Fig 2:**
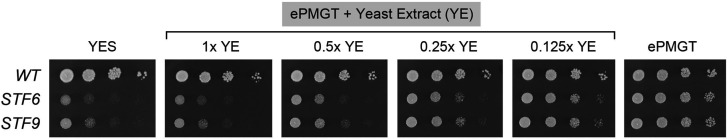
*asp1-STF6* and *STF9* toxicosis is manifest in growth medium containing yeast extract (YE). Serial fivefold dilutions of *S. pombe* wild-type cells and cells bearing the indicated *asp1* alleles were spot tested for growth at 30°C on YES agar, ePMGT (enhanced pombe minimal glutamate plus thiamine) agar, and ePMGT agar supplemented with 1×, 0.5×, 0.25×, or 0.125× yeast extract (where 1× YE is the concentration of YE present in YES medium).

Loss-of-function mutations in the C-terminal Asp1 pyrophosphatase domain exert disparate effects on vegetative growth on YES medium, ranging from benign (active site mutant H397A) to sick (G863D, C643Y, and H686Y) to dead (C-terminal truncations 386-stop and 493-stop) (Fig. 1). The reasons for the differential severity are unclear. We queried whether the apparent lethality of *STF6* (386-stop) and *STF9* (493-stop) progeny of a genetic cross might arise from an inability of single-mutant *STF6* and *STF9* spores to either germinate or undergo post-germination outgrowth. This turned out to be the case, insofar as we were able to recover viable haploid *STF6* and *STF9* progeny after sporulating an *asp1-STF::hygMX/asp1^+^ rpb1-CTD-T4A::natMX*/*rpb1^+^* diploid strain bearing a complementing *asp1^+^ ura4^+^* plasmid, selecting haploids for the marked *STF6* or *STF9* allele, and screening for those that were sensitive to nourseothricin (i.e., lacking *rpb1-CTD-T4A*), all of which were Ura^+^ (i.e., retained the *asp1^+^ ura4^+^* plasmid) and grew well on YES agar. We then recovered *STF6* and *STF9* cells lacking the *asp1^+^ ura4^+^* plasmid by selection for growth on medium containing 5-5-fluoroorotic acid (5-FOA). Spot-testing of serial dilutions of FOA-selected *STF6* and *STF9* cells for growth on YES agar revealed them to be very sick, similar to the sick phenotype displayed by *STF3*, *STF5*, and *STF7* (Fig. 2; Fig. S3A).

To further interrogate the stage at which *asp1-STF6* and *STF9* toxicity to spores is manifest, we crossed *asp1-STF6::hygMX spx1*∆*::ura4^+^* or *asp1-STF9::hygMX spx1*∆*::ura4^+^* strains (in which the *STF* growth defect is suppressed in the absence of Spx1) to an *asp1^+^ spx1-WT::kanMX* strain, selected for diploids via drug resistance markers linked to the alleles of interest, which were then sporulated and analyzed by tetrad dissection and incubation of individual spores on YES agar. Viable spores from each tetrad were genotyped via screening for the *kanMX*, *hygMX*, and *ura4*^+^ markers linked to the alleles of interest. The salient findings (shown in Fig. S1, left panels) were that: (i) tetratype (TT) tetrads formed three viable haploid progeny*—asp1-STF spx1*∆, *asp1^+^ spx1-WT*, and *asp1^+^ spx1*∆—and failed to yield macroscopic colonies from the *asp1-STF spx1-WT* spores; (ii) nonparental ditype (NPD) tetrads generated two viable *asp1^+^ spx1*∆ progeny but no macroscopic colonies from the two *asp1-STF spx1-WT* spores; and (iii) only parental ditype (PD) tetrads yielded four viable spores. Microscopic examination of the *asp1-STF spx1-WT* spores after 5 days of incubation on YES agar (Fig. S2A) indicated that they underwent germination followed by one or more rounds of mitotic growth, either arresting after the two-cell stage or forming microcolonies of varying size. Visualization of the post-germination vegetative cells present in an *asp1-STF* microcolony after resuspension in liquid revealed them to have grossly abnormal morphology (Fig. S2B).

### *asp1-STF* mutant toxicosis is manifest in growth medium containing yeast extract

ePMGT (enhanced pombe minimal glutamate plus thiamine) is a customized synthetic medium that we have deployed for studies of phosphate homeostasis (14). A striking finding was that the *STF6* and *STF9* mutants grew nearly as well as wild-type cells on ePMGT agar at all temperatures tested, i.e., the number of viable colonies was similar at equivalent dilutions, though the *STF6* and *STF9* colony sizes were slightly smaller than wild-type (Fig. 2; Fig. S3A). Moreover, the addition of yeast extract to ePMGT, at the same level present in YES, reinstated the severe sickness seen with YES as the growth medium (Fig. 2). Back-titration of the yeast extract added to ePMGT in twofold decrements resulted in step-wise softening of the toxicity, evinced by the colony densities of the spots on the agar plates. Note that growth of *STF6* and *STF9* was slowed even when one-eighth of the standard amount of yeast extract was included in the ePMGT medium, as judged by colony size (Fig. 2). The severe growth defect of *STF3 (G863D*), *STF5 (C643Y*), and *STF7 (H686Y*) mutants on YES medium was similarly ameliorated when these strains were tested for growth on ePMGT medium (Fig. S3A). We conclude that a titratable component in yeast extract is necessary to exert the toxicity of all five *asp1-STF* strains bearing inactivating mutations in the Asp1 pyrophosphatase domain.

This conclusion was fortified by tetrad analysis of the outcomes of the cross of *asp1-STF6 spx1*∆ or *asp1-STF9 spx1*∆ strains to an *asp1^+^ spx1*-*WT* strain when the individual spores were incubated for 5 days on ePMGT agar. Here, we recovered viable colonies from all four spores derived from TT, NPD, and PD tetrads (Fig. S1, right panels).

### Pho1 is derepressed in *asp1-STF* mutants grown in ePMGT medium

We gauged the impact of the *asp1-STF* alleles on Pho1 protein expression by assaying cell surface Pho1 acid phosphatase activity of wild-type and mutant cells during exponential growth at 30°C in ePMGT medium. The basal Pho1 activity of wild-type cells was derepressed by 15- to 18-fold in the *asp1-STF* cells (Fig. S3B).

### Transcriptional profiling of *STF6* and *STF9* cells grown in ePMGT medium

**Fig 3:**
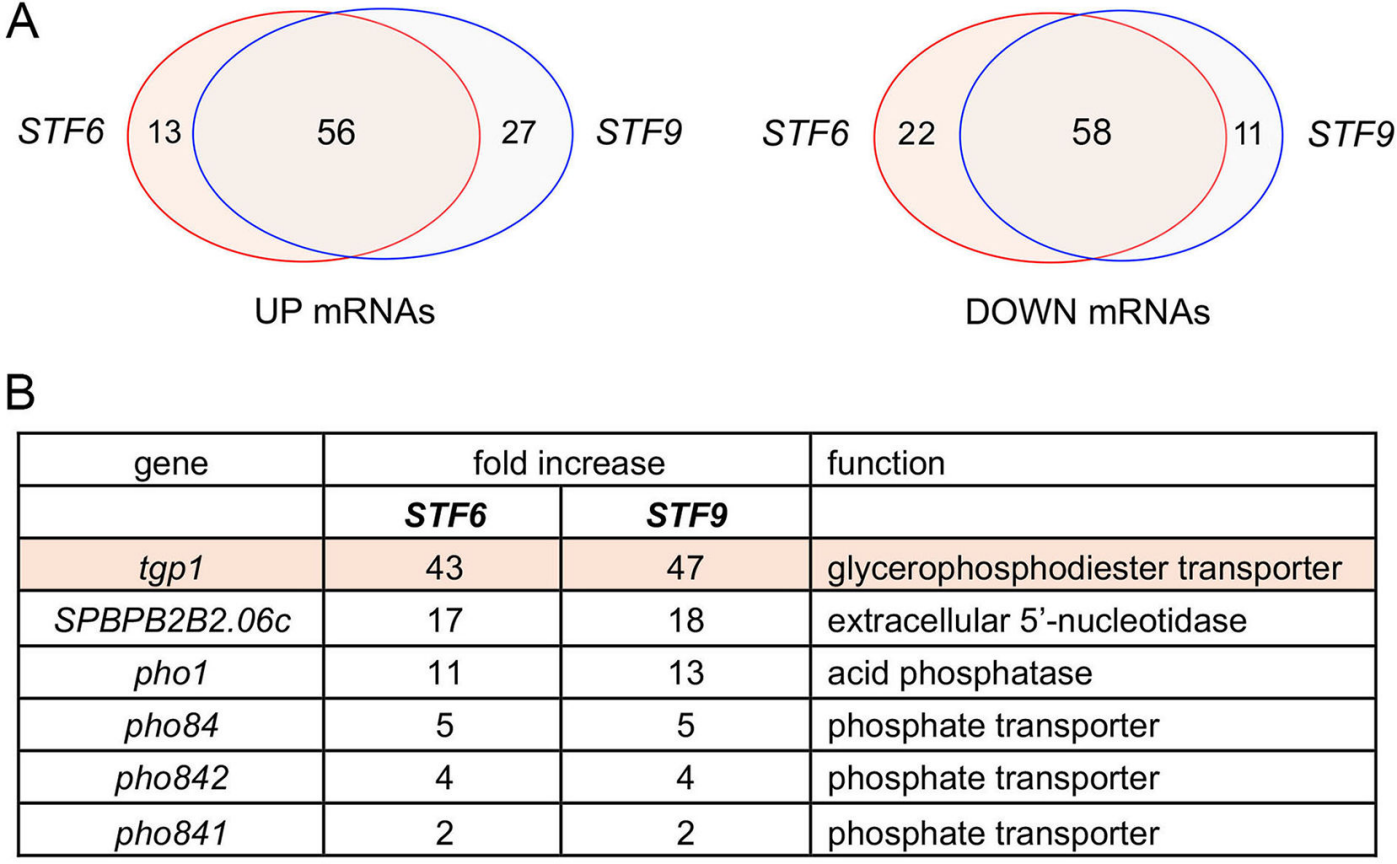
Upregulation of phosphate homeostasis genes in *STF6* and *STF9* cells. (**A**) Venn diagrams showing the number of mRNAs upregulated (UP) or downregulated (DOWN) by ≥2-fold in *STF6* and *STF9* cells and the overlap between the data sets. (**B**) Transcriptome profiling identified six phosphate acquisition genes that were upregulated in *STF6* and *STF9* cells. The upregulated genes are listed according to the fold-increase in transcript level. The function of each gene product is indicated in the right-most column.

We performed RNA-seq on poly(A)^+^ RNA isolated from *STF6* and *STF9* cells and from wild-type controls. cDNAs obtained from three biological replicates (using RNA from cells grown to mid-log phase in ePMGT medium at 30°C) were sequenced for each strain. In the data sets, 90%–97% of the reads were mapped to unique genomic loci (Fig. S4). Read densities (RPKM) for individual genes were highly reproducible between biological replicates (Pearson coefficients of 0.968–0.983; Fig. S4). A cutoff of ±2-fold change in normalized transcript read level and an adjusted *P*-value of ≤0.05 were the criteria applied to derive an initial list of differentially expressed annotated loci in the *STF* mutants versus the wild-type control. We then focused on differentially expressed genes with average normalized read counts ≥100 in either the *STF* or wild-type strains to eliminate transcripts that were expressed at very low levels in vegetative cells. We thereby identified sets of 69 and 83 annotated protein-coding genes that were upregulated by these criteria in *STF6* and *STF9* cells, respectively, of which 56 mRNAs were coordinately upregulated in both *STF* mutants (Fig. 3A; Table S1). The shared upregulated mRNAs included the three *PHO* regulon genes: *tgp1* (up 43- to 47-fold), *pho1* (up 11- to 13-fold), and *pho84* (up 5-fold) (Fig. 3B). The *ecl3* gene, upregulated by 13-fold, is located on chromosome II, adjacent to and in opposite orientation to the *prt2* lncRNA gene of the phosphate-regulated *prt2–pho84–prt–pho1* gene cluster; *ecl3* is co-regulated with the *PHO* genes in several mutant genetic backgrounds and during phosphate starvation (6, 14). Three other genes involved in phosphate acquisition were also upregulated: *SPBPB2B2.06c* encoding an extracellular 5*'* nucleotidase (up 17- to 18-fold); and *pho842* (up 4-fold) and *pho841* (up 2-fold) encoding plasma membrane inorganic phosphate transporters (Fig. 3B). The RNA-seq experiment identified sets of 80 and 69 protein-coding genes that were downregulated by ≥2-fold in *STF6* and *STF9* cells, respectively, 58 of which were coordinately downregulated in both mutants (Fig. 3A; Table S1). Three iron regulon mRNAs*—frp1*, *frp2*, and *abc3*—were downregulated by two- to fourfold. These results, specifically the upregulated genes in *STF6* and *STF9* cells, fortify prior RNA-seq data in establishing that perturbations of IP_8_ dynamics affect the expression of genes involved in phosphate homeostasis, many of which are known to be sensitive to manipulation of 3*'*-processing and transcription termination factors.

### Spontaneous suppressors of *STF6* and *STF9* toxicosis

**Fig 4:**
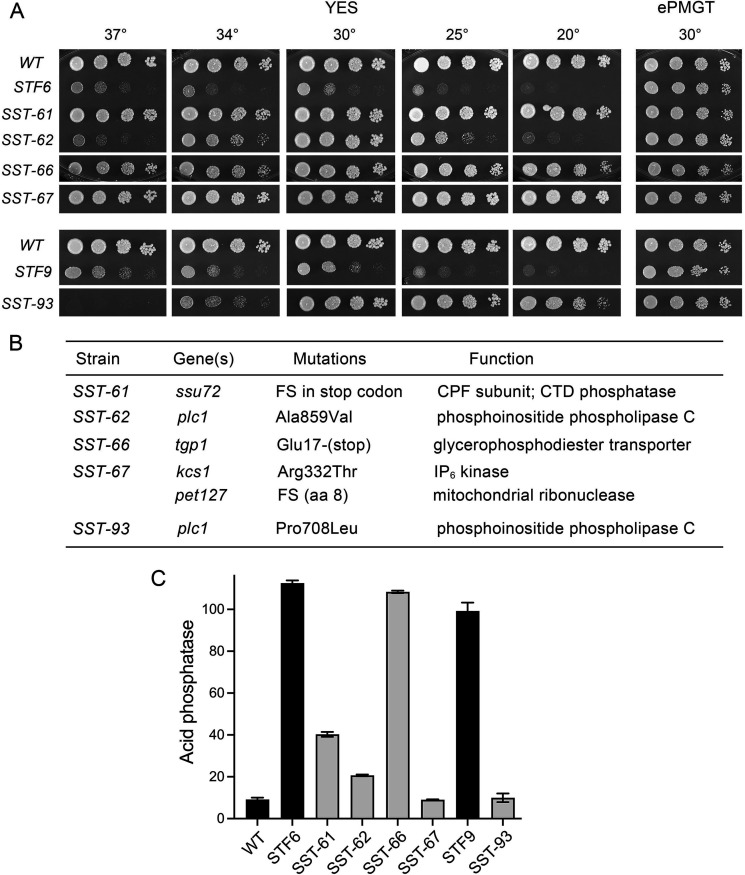
Isolation of spontaneous suppressors of *STF6* and *STF9* toxicosis. (**A**) Serial fivefold dilutions of fission yeast strains (as specified on the left) were spot tested for growth on YES agar at the indicated temperatures and on ePMGT agar at 30°C. (**B**) Whole-genome sequencing of the *SST* strains revealed the indicated mutations. (**C**) The indicated strains were grown to *A*_600_ of 0.5–0.8 in liquid culture in ePMGT medium at 30°C. Cells were then harvested, washed with water, and assayed for Pho1 acid phosphatase activity.

The newfound ability to interrogate *STF6* and *STF9* under permissive (ePMGT) versus restrictive (YES) conditions sets the stage for dissecting the basis for IP_8_ toxicosis via genetic suppression. We screened for candidate *SST* mutants by plating *STF6* and *STF9* cells on YES agar at 30°C and selecting rare single colonies that grew to large size against a background of tiny colonies. These were grown and re-streaked for single colonies, which were homogeneously larger than the respective parental *STF* strains. We report here four independent suppressors of *STF6*, named *SST-61*, *SST-62*, *SST-66*, and *SST-67*, and one suppressor of *STF9*, named *SST-93*. The *SST-61* and *SST-67* strains grew as well as wild-type on YES agar at all temperatures (Fig. 4A). The *SST-66* strain formed slightly smaller colonies than wild-type at all temperatures. *SST-62* and *SST-93* cells grew well at 30°C but displayed *ts* and *cs* growth defects at 37°C and 20°C (Fig. 4A). All the *SST* strains grew as well as wild-type on ePMGT medium at 30°C (Fig. 4A). Assay of the *SST* strains for cell surface Pho1 acid phosphatase activity during exponential growth at 30°C in ePMGT medium showed that Pho1 activity was reduced to near wild-type levels in *SST-67* and *SST-93* cells and dampened by fivefold in *SST-62* cells or threefold in *SST-61* cells vis-à-vis the parental *STF6* strain (Fig. 4C). Pho1 activity remained fully derepressed in *SST-66* cells compared to the original *STF6* strain (Fig. 4C).

### Diverse mutations suppress *STF6* and *STF9* toxicosis

Paired-end Illumina sequencing of unamplified genomic DNA from the *SST* strains was performed to achieve at least 100-fold coverage of the fission yeast genome. The *SST-6* and *SST-9* genomes were compared to those of the parental *STF6* and *STF9* strains. All the *SST* isolates retained their original *asp1-STF6* or *STF9* alleles. The mutations identified for each isolate are compiled in Fig. 4B. The mutated proteins fall into several functional categories: (i) Ssu72, a CTD phosphatase subunit of CPF; (ii) upstream enzymes of inositol pyrophosphate biogenesis, Plc1 and Kcs1; and (iii) glycerophosphodiester transporter Tgp1, a *PHO* regulon component protein that is transcriptionally derepressed in *STF6* and *STF9* cells.

### CPF subunit Ssu72

Strain *SST-61* contains a single −1 frameshift mutation in the stop codon of the *ssu72* gene, the effect of which is to extend the open reading frame and append a 25-aa peptide segment (NIIYRYTSLSNLIKHVLSPSISTTF*) to the C-terminus of the native 197-aa Ssu72 polypeptide. Because previous studies established that: (i) the lethality of *STF6* and *STF9* on YES medium was suppressed by a purposefully engineered phosphatase-dead C13S active site mutation of Ssu72 (10) and (ii) that the synthetic near-lethality of *asp1-H397A rad24*∆ was suppressed by a spontaneous mutation *ssu72-(Q57-stop*) that ablated the Ssu72 protein (15), we surmise that the alien C-terminal extension to Ssu72 in the *SST-61* strain results in loss of Ssu72 function. The isolation of this *ssu72* allele in a screen for spontaneous suppressors of *STF6* affirms the role of the Ssu72 CTD phosphatase subunit of CPF as a mediator of IP_8_ toxicosis.

### Phospholipase C Plc1

Strain *SST-62* contains a missense mutation (A859V) in the *plc1* gene encoding phosphoinositide phospholipase C, an enzyme that hydrolyzes membrane phosphoinositide PIP_2_ (1-phosphatidylinositol 4,5-bisphosphate) to release IP_3_ (inositol-1,4,5-trisphosphate) and diacylglycerol, thereby providing the precursor for the synthesis of IP_6_ and its subsequent conversion to inositol pyrophosphates (16). Strain *SST-93* contains a different missense mutation (P708L) in the *plc1* gene (Fig. 4B). Both *plc1* mutations elicit *ts/cs* growth phenotypes in the *asp1-STF* strain backgrounds (Fig. 4A), and both substantially blunt the Pho1 derepression characteristic of the parental *STF6* and *STF9* strains (Fig. 4C). The 899-aa Plc1 protein is composed of a pleckstrin homology (PH) domain, tandem EF hand domains, a bipartite phosphodiesterase catalytic domain, and a C2 domain. According to Alpha Fold (https://alphafold.ebi.ac.uk/entry/P40977), the Ala859 residue is located on the hydrophobic face of a β-strand of the C2 domain; its mutation to valine would introduce steric clash with nearby aliphatic side chains. Pro708 is located at the beginning of an α-helix in the catalytic domain; its mutation to leucine would engender steric clash with neighboring structural elements.

**Fig 5:**
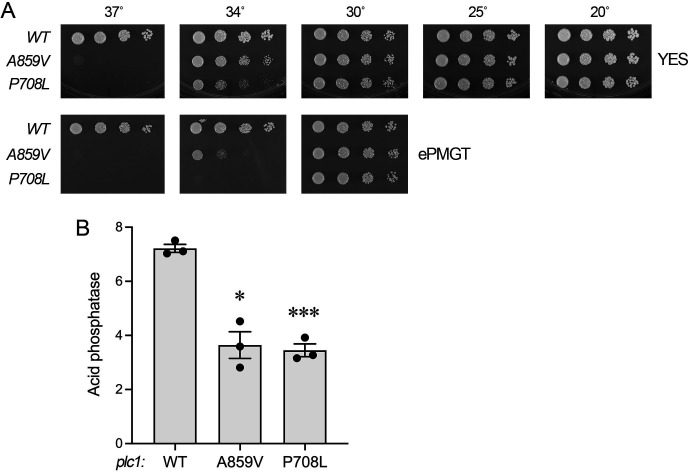
Effect of *plc1-A859V* and *plc1-P708L* mutations on fission yeast growth and Pho1 expression. (**A**) Serial fivefold dilutions of fission yeast strains (as specified on the left) were spot tested for growth on YES agar and ePMGT agar at the indicated temperatures. (**B**) The indicated strains grown in YES medium were assayed for Pho1 acid phosphatase activity. Pairwise *t*-tests showed significant differences between wild-type and *plc1* mutants (*P*-values of 0.013 and 0.0005 for wild-type versus *A859V* and *P708L*, respectively, denoted by asterisks).

To gauge whether *plc1-A859V* and *plc1-P708L* elicit a phenotype in a wild-type genetic background, the *SST-62* and *SST-93* strains, in which the *STF6* and *STF9* alleles are marked with a downstream *hygMX* cassette, were crossed to an *asp1^+^* strain. Viable haploid progeny that grew on YES agar were tested by replica plating for growth on hygromycin-containing medium. Hygromycin-sensitive strains (i.e., harboring the *asp1^+^* allele) were then screened for the *plc1-A859V* and *plc1-P708L* mutations by a one-step PCR assay for SNP detection (17) and confirmed by sequencing the relevant PCR-amplified segment of the *plc1* ORF. The *plc1-A859V* and *plc1-P708L* cells grew as well as wild-type on YES agar at 20°C, 25°C, and 30°C, were slow growing at 34°C and failed to form colonies at 37°C (Fig. 5A). The *plc1-A859V* and *plc1-P708L* strains displayed the same *ts* defect when tested for growth on ePMGT medium (Fig. 5A). Because a *plc1*∆ null mutant similarly grows well on YES at 30°C but fails to grow at 37°C (18, 19), we surmise that *plc1-A859V* and *plc1-P708L* are mutations that diminish phospholipase C activity, which could ameliorate the toxicity of *asp1-STF* by partly stemming the flux of IP_3_ toward inositol pyrophosphate synthesis. Assay of Pho1 acid phosphatase activity showed that Pho1 expression was hyper-repressed in *plc1-A859V* and *plc1-P708L* cells compared to a wild-type control (Fig. 5B). Hyper-repression of Pho1 is a feature of *asp1*∆ cells that cannot synthesize IP_8_ (6).

### IP_6_ kinase Kcs1

**Fig 6:**
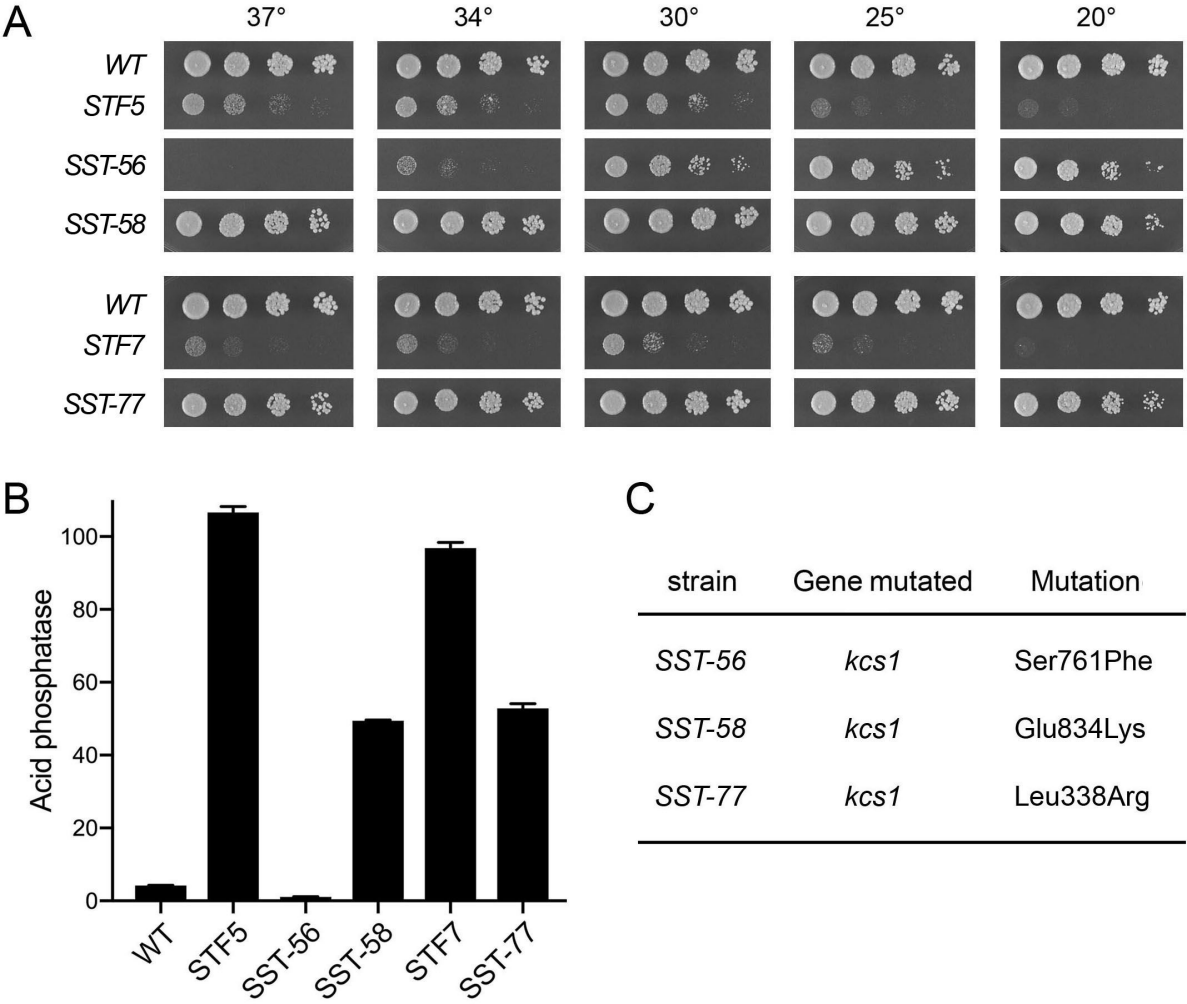
Screen for suppression of *STF5* and *STF7* toxicosis yields additional *kcs1* mutant alleles. (**A**) Serial fivefold dilutions of fission yeast strains (as specified on the left) were spot tested for growth on YES agar at the indicated temperatures. (**B**) The indicated strains grown in YES medium were assayed for Pho1 acid phosphatase activity. (**C**) Whole-genome sequencing of the *SST* strains revealed the indicated *kcs1* mutations.

Strain *SST-67* contains a missense mutation (*R332T*) in the essential *kcs1* gene encoding the kinase enzyme that converts IP_6_ to 5-IP_7_. (*SST-67* contains a second incidental frameshift mutation at codon 8 of the inessential *pet127* gene encoding a 524-aa putative mitochondrial ribonuclease.) In a parallel screen for suppressors of the severe growth defect of *asp1-STF5* and *asp1-STF7* strains on YES medium, we recovered two independent suppressors, named *SST-58* and *SST-77*, that restored near wild-type growth on YES at all temperatures (Fig. 6A). A third suppressor, *SST-56*, displayed improved growth at 25°C and 20°C compared to *STF5* but was unable to grow at 37°C (Fig. 6A). Whole-genome sequencing of the suppressed strains and comparison to *STF5* and *STF7* revealed unique missense mutations in the *kcs1* gene in each case: *S761F* in *SST-56, E834K* in *SST-58*, and *L338R* in *SST-77* (Fig. 6C). The fact that four different missense mutants of Kcs1 kinase were recovered as suppressors of an Asp1 pyrophosphatase loss-of-function phenotype suggests that they do so by limiting the production of 5-IP_7_, the substrate for Asp1 kinase. Assay of these three *SST* strains for cell surface Pho1 acid phosphatase activity during growth at 30°C in YES medium showed that the *S761F* mutation effaced the derepression of Pho1 in the original *STF5* strain, whereas the *E834K* and *L338R* mutations reduced Pho1 expression by about half compared to *STF5* and *STF7*, respectively (Fig. 6B).

**Fig 7:**
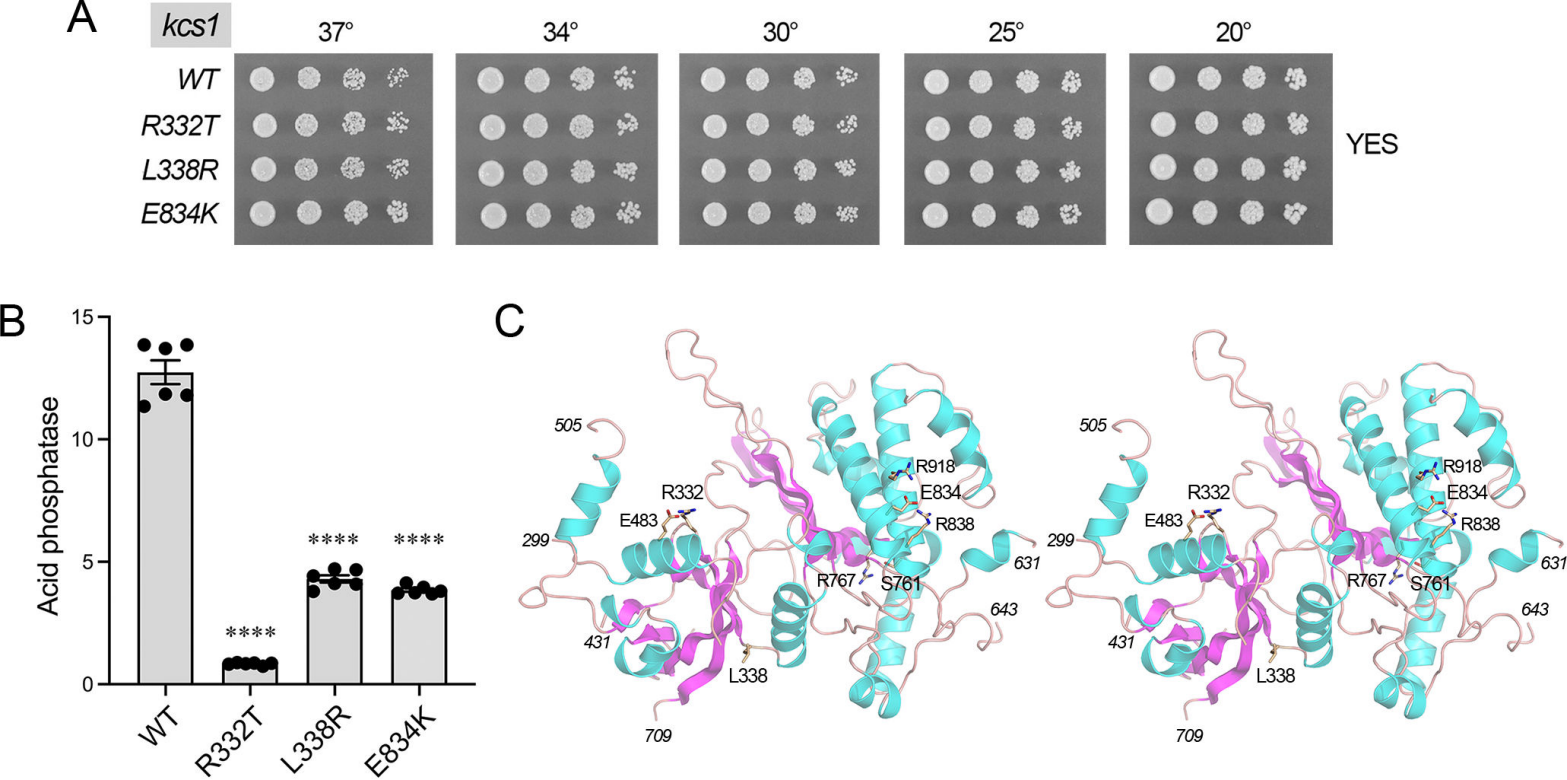
Effect of *kcs1* mutations on fission yeast growth and Pho1 expression. (**A**) Growth of wild-type and mutant *kcs1* strains (as specified on the left) was assessed by spotting serial dilutions of cells to YES agar at the indicated temperatures. (**B**) Acid phosphatase activities of cells with the indicated *kcs1* alleles. Unpaired Welch’s *t*-tests of the data for wild-type versus *kcs1* mutants differed significantly (*P*-value <0.0001, denoted by ****). (**C**) Stereo view of the Kcs1 kinase tertiary structure predicted by Alpha Fold, with the indicated amino acid side chains shown as stick models.

Because the *kcs1* and *asp1* genes are linked on chromosome III (70 kbp apart), and the *kcs1* suppressor alleles in the *SST* strains are unmarked, we could not rely on crossing to readily obtain the *kcs1* mutants in an *asp1-WT* background. Thus, we replaced one of the chromosomal *kcs1*^+^ loci in a diploid *S. pombe* strain with *kcs1–kanMX* cassettes, comprising *kcs1-WT*, *kcs1-R332T*, *kcs1-L338R*, *kcs1-E834K*, or *kcs1-S761F* driven by the endogenous *kcs1* promoter and flanked by an *ADH1* terminator and a downstream *kanMX* marker. Viable G418-resistant *kcs1-WT–kanMX*, *kcs1-R332T–kanMX*, *kcs1-L338R–kanMX*, and *kcs1-E834K–kanMX* haploids were recovered after sporulation. However, we failed to recover a G418-resistant *kcs1-S761F–kanMX* strain, signifying that the *S761F* mutation was lethal in a wild-type *asp1*^+^ genetic background. The 3*'*-marked *kcs1-WT*, *kcs1-R332T*, *kcs1-L338R*, and *kcs1-E834K* strains grew equally well on YES agar at all temperatures tested (Fig. 7A). Assay of acid phosphatase activity showed that Pho1 expression was hyper-repressed in *kcs1-R332T*, *kcs1-L338R*, and *kcs1-E834K* cells compared to an identically marked *kcs1-WT* control (Fig. 7B).

An Alpha Fold model of the 967-aa Kcs1 protein (https://alphafold.ebi.ac.uk/entry/O74561) predicts that much of the polypeptide consists of intrinsically disordered segments, i.e., aa 1–298, 375–410, 431–471, 506–630, 648–709, and 955–967. Alpha Fold predicts that the remainder of the Kcs1 polypeptide adopts an inositol polyphosphate kinase fold (shown in stereo in Fig. 7C) similar to *Entamoeba histolytica* IP6K/IP3K (20). It is noteworthy that all four of the *STF*-suppressive *SST* mutations of Kcs1 are located within the structurally ordered kinase fold. Ser761, mutation of which to Phe causes a severe *ts* growth defect in the *SST-56* context and is lethal in a wild-type background, is predicted to engage in a hydrogen bond to Arg767 (Fig. 7C). The Ser761-Arg767 pair is conserved as Thr128-Arg134 in *Entamoeba* IP6K/IP3K, where Arg134 forms part of the IP_6_-binding pocket. Installing Phe in lieu of Ser would be predicted to impose a steric clash adjacent to the substrate-binding site that could account for the *kcs1-S761F* growth defects. Glu834 anchors a predicted salt-bridge network to Arg838 and Arg918 (Fig. 7C) that would be severed by its mutation to Lys. Arg332 is predicted to make a salt bridge to Glu483 (Fig. 7C) that would be lost when it is changed to Thr. Leu338 is predicted to make van der Waals interactions with Val336, Asn343, and Ile716, such that its replacement by Arg might impose a clash that impacts the local fold.

### Transcriptional profiling of *kcs1* mutants

**Fig 8:**
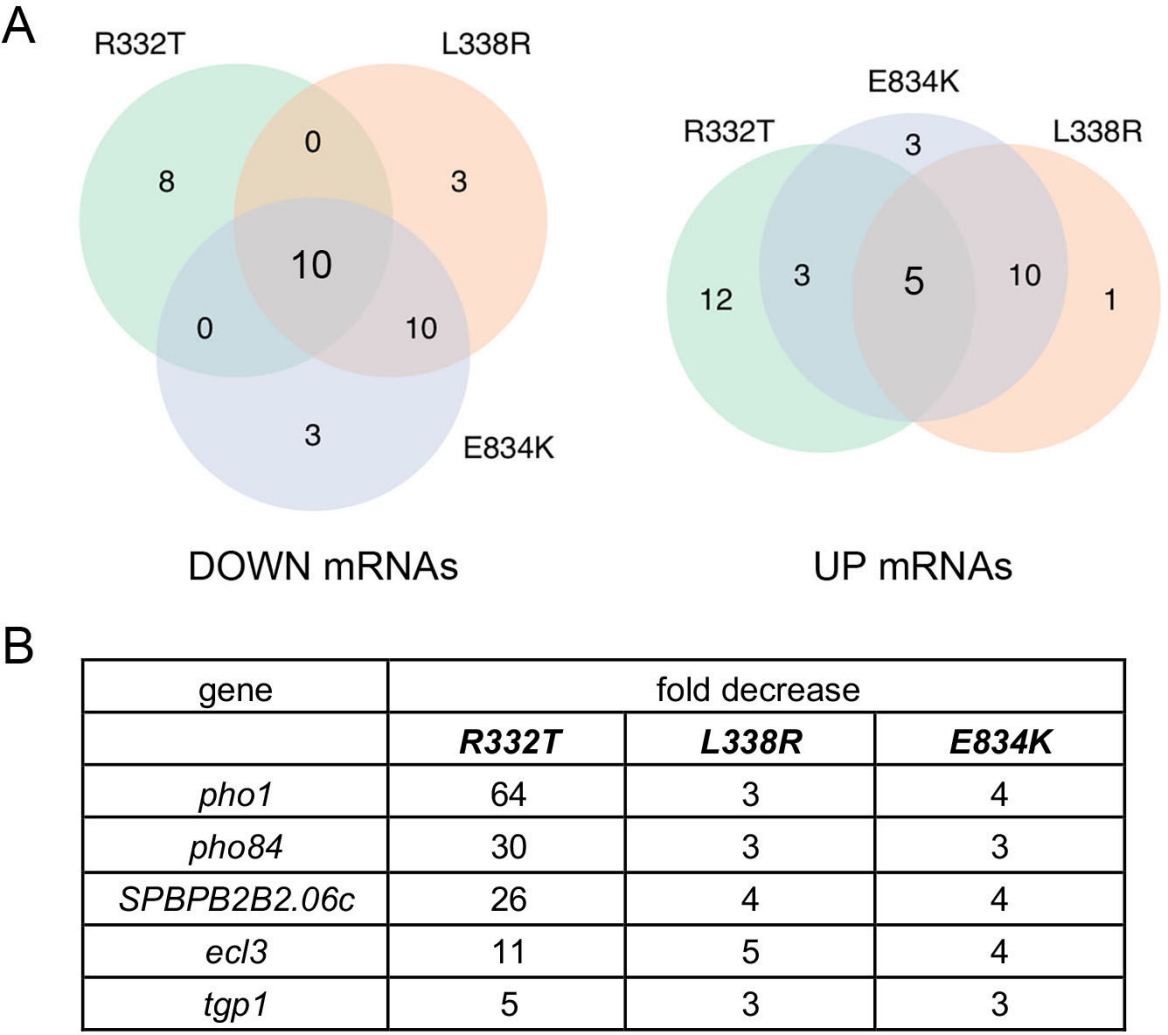
Transcriptional profiling of *kcs1* mutants. (**A**) Overlaps of protein-coding genes that were downregulated (DOWN) or upregulated (UP) by ≥2-fold in the indicated *kcs1* mutants compared to wild-type. (**B**) List of five phosphate-responsive genes that were coordinately downregulated in the indicated *kcs1* mutants. The fold decrease in transcript level is shown.

We performed RNA-seq on poly(A)^+^ RNA isolated from *kcs1-R332T*, *kcs1-L338R*, and *kcs1-E834K* cells and from wild-type control cells. cDNAs obtained from three biological replicates (using RNA from cells grown to mid-log phase in YES medium at 30°C) were sequenced for each strain. In the data sets, 99% of the reads were mapped to unique genomic loci (Fig. S6). Read densities (RPKM) for individual genes were highly reproducible between biological replicates (Pearson coefficients of 0.970–0.985; Fig. S6). Applying the same criteria used for RNA-seq analysis of *STF6* and *STF9* cells, we identified sets of 18, 23, and 23 annotated protein-coding genes that were downregulated by ≥2-fold in *kcs1-R332T*, *kcs1-L338R*, and *kcs1-E834K* cells, respectively, of which 10 mRNAs were coordinately downregulated in all three *kcs1* mutants (Fig. 8A; Table S2). The shared downregulated mRNAs included the phosphate-responsive genes *tgp1*, *pho1*, *pho84*, *SPBPB2B2.06c*, and *ecl3* (Fig. 8B). Consistent with the greater degree of hyper-repression on Pho1 activity by *kcs1-R332T* versus *kcs1-L338R* and *kcs1-E834K* (Fig. 7B), the *kcs1-R332T* allele exerted a greater impact on *pho1* mRNA level (64-fold decrement) than did *kcs1-L338R* and *kcs1-E834K* (Fig. 8B). RNA-seq identified 20, 16, and 21 mRNAs that were upregulated in *kcs1-R332T*, *kcs1-L338R*, and *kcs1-E834K* cells, respectively, of which 5 were upregulated in all three *kcs1* mutants (Fig. 8A). Five iron regulon genes*—str1*, *srx1*, *fip1*, *frp1*, and *fio1*—were upregulated by two- to fourfold in *kcs1-R332T* cells. *srx1* and *fip1* expression were increased twofold in *kcs1-L338R* and *kcs1-E834K* cells (Table S2).

### Glycerophosphodiester transporter Tgp1

Strain *SST-66* contains an inactivating nonsense mutation at codon 18 in the *tgp1* gene encoding a 528-aa polypeptide homologous to *Saccharomyces cerevisiae* Git1 and *Candida albicans* paralogs CaGit1, 2, 3, 4. The Git-family proteins function as transmembrane transporters of extracellular glycerophosphocholine (GPC) and/or glycerophosphoinositol (GPI) (21–25). *S. cerevisiae* Git1 is much more active in driving uptake of ^3^H-GPI versus ^3^H-GPC (24). The *Candida* paralogs display specificity for either GPI (Git1) or GPC (Git3 and Git4) (24, 25). To our knowledge, the transport activity and substrate specificities of fission yeast Tgp1 have not been reported. Nonetheless, it is well established that *tgp1* is part of a three-gene *PHO* regulon that is repressed under phosphate-replete conditions and derepressed during phosphate starvation (14, 26), presumably to import glycerophosphodiesters as a source of phosphate for phosphate-starved cells (22, 27). The *tgp1* gene is repressed in phosphate-replete cells by *nc-tgp1* lncRNA-mediated transcriptional interference (7, 28, 29). *tgp1* is derepressed in phosphate-replete conditions by inactivating mutations of Asp1 pyrophosphatase, whereby increased IP_8_ levels elicit precocious termination of interfering lncRNA transcription (6).

### Glycerophosphocholine recapitulates the toxicity of yeast extract to *STF6* and *STF9* cells

**Fig 9:**
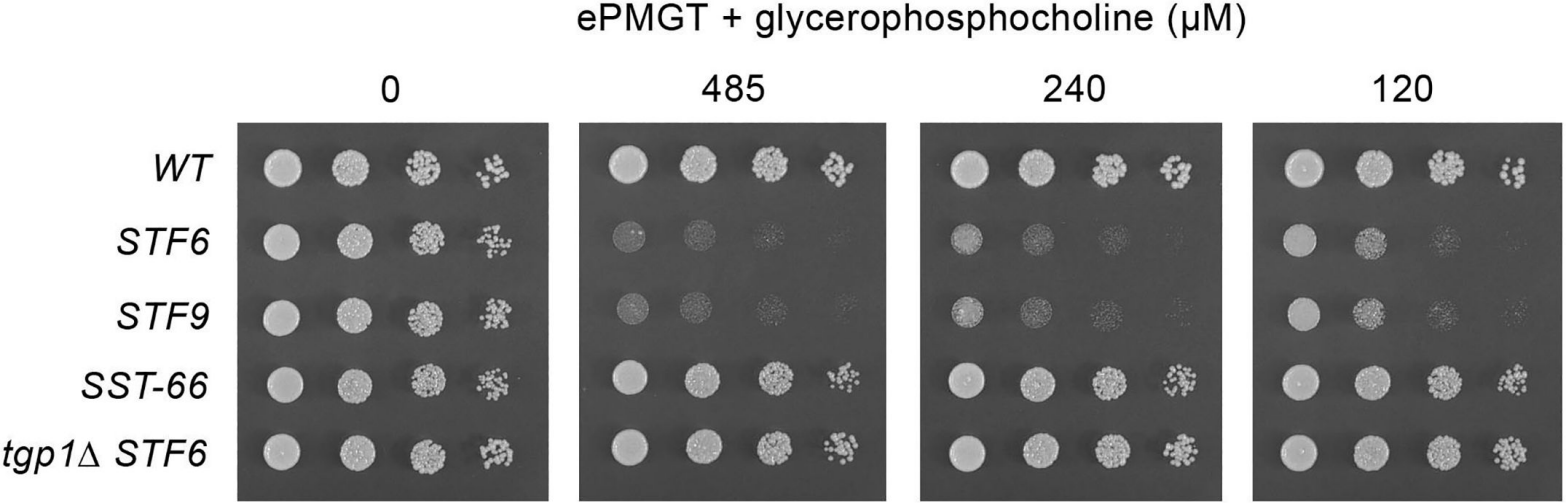
Glycerophosphocholine recapitulates the toxicity of yeast extract to *STF6* and *STF9* cells. Serial dilutions of the yeast strains indicated at the left were spot tested for growth on ePMGT agar without or with GPC as specified at the top. The plates were photographed after 3 days of incubation at 30°C.

The findings here that (i) *tgp1* mRNA levels are increased by ~45-fold in *STF6* and *STF9* cells; (ii) growth of *STF6* and *STF9* cells is inhibited by a titratable constituent of yeast extract; and (iii) the severe growth defect of *STF6* cells on YES medium is suppressed completely by a null allele of *tgp1* prompt a hypothesis that overexpression of Tgp1 in *STF6* and *STF9* cells results in the increased import of a component of yeast extract to levels that are toxic to *STF6* and *STF9*. Pairwise BLAST alignments of the Tgp1 amino acid sequence to those of the functionally characterized *Saccharomyces* and *Candida* Git-family homologs indicated that Tgp1 is more closely related to CaGit3 (204 positions of side chain identity) and CaGit4 (197 identities) that transport GPC than it is to *S. cerevisiae* Git1 (149 identities) or CaGit1 (157 identities) that is selective for GPI transport. Accordingly, we queried whether supplementation of ePMGT with GPC might mimic the growth suppressive effect of yeast extract on *STF6* and *STF9* cells that overexpress *tgp1*. As shown in Fig. 9, this was indeed the case in the presence of 120, 240, or 485 µM GPC, concentrations that had no effect on the growth of wild-type cells that do not overexpress *tgp1*. Moreover, the toxicity of GPC to *STF6* was suppressed by the *tgp1-*disruptive nonsense allele in the *SST-66* strain and by an engineered *tgp1*∆ allele in which the *nc-tgp1–tgp1* locus was deleted in its entirety (Fig. 9). Additional controls established that ePMGT supplementation with choline (up to 4 mM) or glycerol-3-phosphate (up to 4 mM) had no deleterious impact on the growth of *STF6* or *STF9* cells (Fig. S5). We surmise that: (i) Tgp1 is a *bona fide* GPC transporter, and (ii) excess intracellular GPC accumulation via overexpressed Tgp1 is somehow toxic to *STF6* and *STF9* cells.

### Overexpression of *tgp1* is toxic to wild-type and *asp1*∆ cells

**Fig 10:**
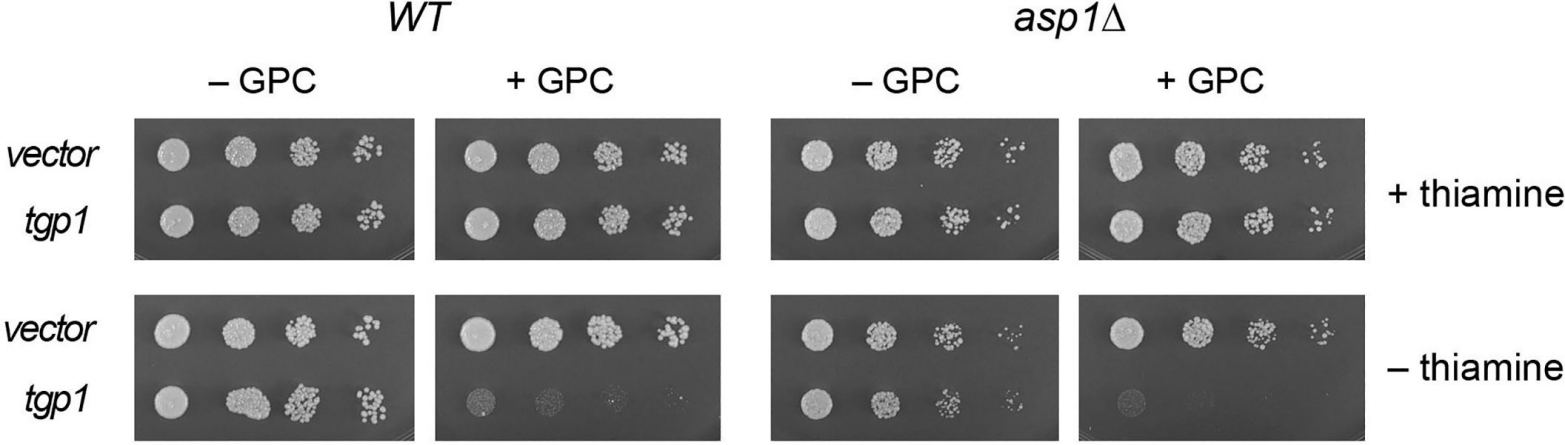
High-copy overexpression of *tgp1* is toxic to wild-type and *asp1*∆ cells. Serial dilutions of *asp1-WT* and *asp1∆* strains bearing the indicated plasmids (as specified on the left) were spot tested for growth on ePMG (-leu) agar or ePMGT (-leu) agar, with or without 0.77 mM GPC. The plates were photographed after 3–4 days of incubation at 30°C.

We constructed a fission yeast multicopy *LEU2* plasmid in which *tgp1* is expressed under the control of the thiamine-repressible *nmt1-41x* promoter. The *tgp1* plasmid and empty vector control were introduced into wild-type cells (with normal levels of IP_8_) and into *asp1*∆ cells (that have no IP_8_). Cells grown to mid-log phase in liquid ePMGT(-leu) medium were harvested, washed with water, and resuspended in water at equivalent *A*_600_. Serial fivefold dilutions were then spotted on ePMGT(-leu), ePMG(-leu), ePMGT(-leu) plus GPC, and ePMG(-leu) plus GPC agar plates, which were incubated at 30°C. The salient findings were that (i) the *nmt1–tgp1* gene inhibited the growth of wild-type and *asp1*∆ cells on thiamine-free ePMG medium containing GPC but not on thiamine-free medium lacking GPC, and (ii) the *nmt1–tgp1* gene did not inhibit the growth of wild-type or *asp1*∆ cells on thiamine-replete ePMGT medium containing GPC (Fig. 10). Thus, overproduction of Tgp1 suffices to elicit GPC toxicosis independent of genetic perturbation of IP_8_ catabolism (as in the *asp1-STF* mutants) and independent of IP_8_ synthesis by Asp1 kinase.

## DISCUSSION

The lncRNA-mediated transcription interference that underlies the repression of the fission yeast *PHO* regulon has afforded a sensitive readout of genetic influences on 3*'*-processing/termination and a powerful tool for discovery of agents and regulators of these steps of the Pol2 transcription cycle. A key insight was that excess IP_8_ derepresses the *PHO* regulon in phosphate-replete cells via its action as an agonist of precocious lncRNA 3*'*-processing/termination (6). Because mutations of IP_8_-catabolizing pyrophosphatases, *per se* or in combination, were either lethal or severely growth defective, it was surmised that accumulation of supra-threshold levels of IP_8_ is toxic to fission yeast (10). Epistasis tests and an initial round of “*SST*” screening for spontaneous suppressors of the growth defects of *asp1-STF* alleles highlighted two ways of ameliorating IP_8_ toxicosis: (i) by mutations of components of the 3*'*-processing/termination machinery that dampen the impact of toxic IP_8_ levels on Pol2 termination and (ii) by mutations of proteins Spx1, Gde1, and Vtc4, each of which contains an SPX domain that acts as an inositol pyrophosphate sensor (10–12).

### Suppression of toxic Asp1 alleles by mutations in Plc1 and Kcs1

**Fig 11:**
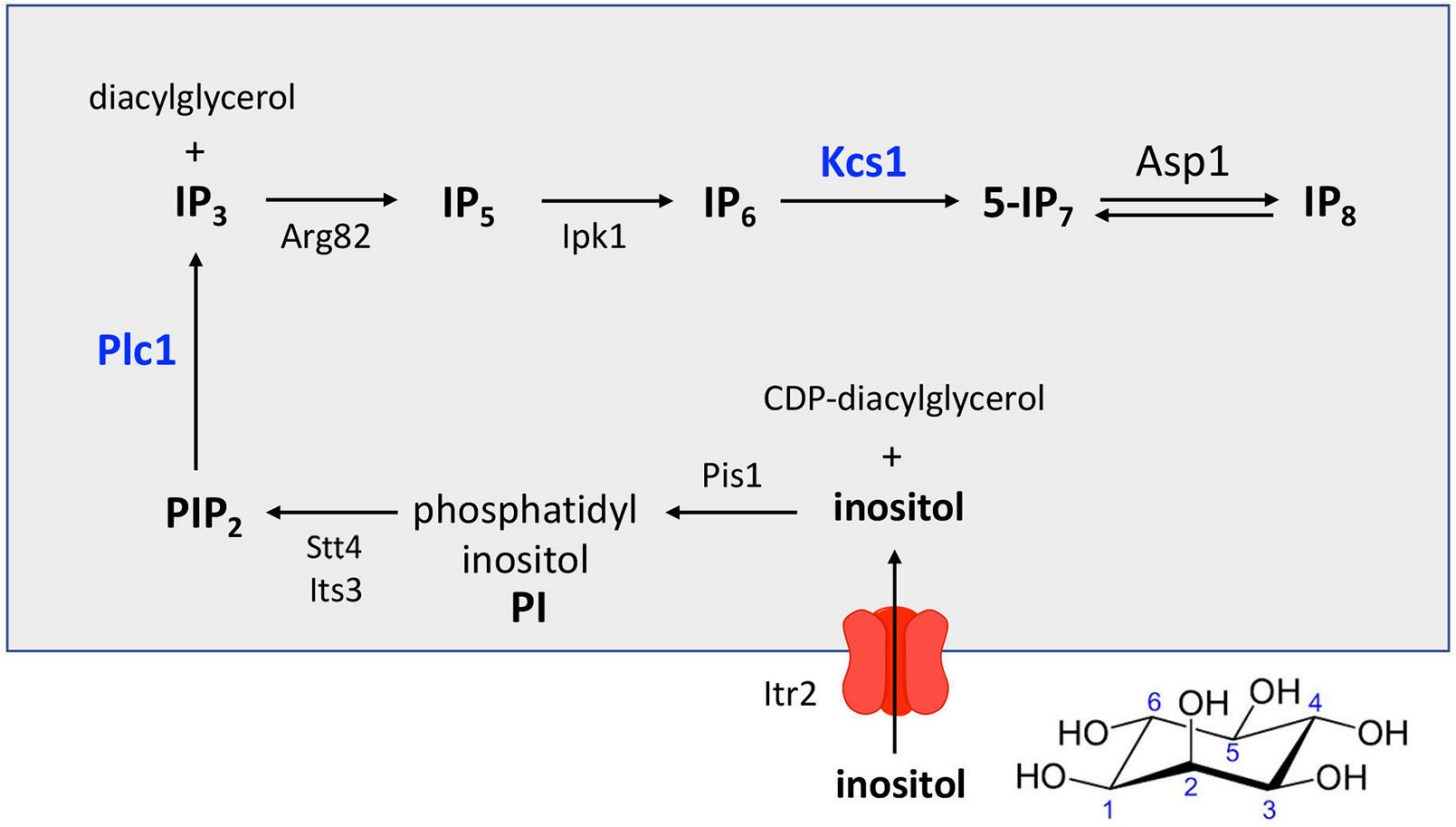
Fission yeast pathway of IP_8_ synthesis starting with uptake of extracellular inositol. See Discussion for details.

Here, by expanding the *SST* suppressor screen, we fulfill an earlier speculation (11) that mutations in upstream enzymes of inositol polyphosphate metabolism might constrain inositol pyrophosphate synthesis by Asp1 kinase so as to blunt the increase in IP_8_ levels when Asp1 pyrophosphatase is crippled, thereby reversing the growth defect of *asp1-STF* mutants. The pathway leading to IP_8_ synthesis is depicted in Fig. 11. *S*. *pombe* is an inositol auxotroph. Inositol taken up from the culture medium by the essential transmembrane transporter Itr2 is converted to phosphatidyl inositol (PI) by the essential CDP-diacylglycerol–inositol 3-phosphatidyltransferase enzyme Pis1. PI is then phosphorylated at positions 4 and 5 of the inositol ring by essential kinases Stt4 and Its3 to generate PIP_2_. Phospholipase C Plc1 action on PIP_2_ liberates IP_3_ (inositol-1,4,5-trisphosphate), which is then converted to IP_5_ (inositol-1,3,4,5,6-pentakisphosphate) by the essential kinase Arg82. IP_5_ is converted to IP_6_ by the kinase Ipk1, thereby providing the substrate for subsequent formation of inositol pyrophosphates by Kcs1 and Asp1. Our *SST* screens yielded two independent missense alleles of *plc1* and four independent missense alleles of *kcs1*.

The genetics of Plc1 and Kcs1 are relatively understudied. Fission yeast with either a *plc1*∆ null mutation or a *plc1-1* allele (molecular lesion unknown) is unable to grow on synthetic medium and displays a *ts* phenotype on rich medium (18, 19, 30). The two *plc1* mutants characterized here*—A859V* and *P708L*—display tight *ts* growth defects on YES and ePMGT media, from which we surmise that they are defective hypomorphs, presumably with reduced enzymatic activity and, hence, reduced flux of IP_3_ toward IP_8_ synthesis.

Deletion of *kcs1* is lethal in fission yeast, and there are no reports in Pombase of other *kcs1* mutant alleles or *kcs1* genetic interactions. Three of the four *kcs1* missense mutants recovered in our screens*—R332T*, *L338R*, and *E834K*—completely reversed the *asp1-STF* growth defect. The *R332T*, *L338R*, and *E834K* mutants were fully viable in an *asp1*^+^ background with respect to growth on YES medium, though they hyper-repressed Pho1 expression, with *R332T* having the strongest effect. This scenario is consistent with a reduction in kinase activity of the mutant Kcs1 enzymes sufficient to limit the pool of 5-IP_7_ available for conversion to IP_8_ by the pyrophosphatase-defective Asp1-STF kinase. By contrast, the *kcs1-S761F* allele was lethal in an *asp1*^+^ background, signifying a more severe defect in 5-IP_7_ synthesis, and the effects of which permitted only partial recovery of growth (at ≤30°C) of the *SST-56* strain bearing *asp1-STF5* and *kcs1-S761F* alleles. The erasure of the increase in Pho1 expression in the *asp1-STF5* mutant by *kcs1-S761F* (to less than the wild-type control) would suggest that IP_8_ fails to accumulate in the *SST-56* strain.

Our suite of three missense *kcs1* alleles allowed us to interrogate the impact of Kcs1 on gene expression system-wide. These hypomorphic *kcs1* mutations elicited dysregulation of a limited number of mRNAs, most notably the downregulation of the same set of phosphate-responsive genes that were upregulated in *STF6* and *STF9* cells (Fig. 3B and 8B). The set of phosphate-responsive mRNAs downregulated in the *kcs1* mutants overlaps the transcripts downregulated in *asp1-D333A* cells that lack the Asp1 kinase activity responsible for conversion of 5-IP_7_ to 1,5-IP_8_ (6).

### Tgp1 is the target gene responsible for IP_8_ toxicosis of the *asp1-STF* mutants in YES medium

Nontoxic mutations affecting IP_8_ catabolism derepress the *PHO* regulon (6). Because the *PHO* genes are not essential for fission yeast growth, it was assumed that the toxicity of *asp1-STF* mutations was caused by dysregulation of expression of one or more essential genes (10). Thus, it was surprising and ultimately highly informative that we: (i) found that the growth defects of the *asp1-STF* mutants were manifest in medium containing yeast extract but not in a synthetic medium ePMGT and (ii) identified a loss-of-function nonsense mutation in *tgp1* as a suppressor of the growth defect of *asp1-STF6*, a strain in which *tgp1* expression is increased 43-fold by virtue of IP_8_-driven precocious termination of the upstream *nc-tgp1* lncRNA that interferes with the *tgp1* mRNA promoter. Unlike the *ssu72*, *plc1*, and *kcs1 SST* mutations, which diminished or eliminated the Pho1 derepression characteristic of the parental *asp1-STF6* and *asp1-STF9* strains, the *tgp1-(17-stop*) allele did not reduce Pho1 expression, indicating that the IP_8_-driven precocious termination of *prt* lncRNA synthesis that underlies Pho1 derepression remained intact in the absence of Tgp1. This implicates Tgp1 overexpression as the cause of toxicity of the *asp1-STF* mutants on YES medium.

### Tgp1-dependent toxicity is mediated by glycerophosphocholine

Supplementing ePMGT with yeast extract sufficed to recapitulate the growth defects of *asp1-STF* mutants on YES medium. Rather than fractionating yeast extract to identify the putative toxicity factor, we followed the genetic clues pointing to the putative glycerophosphodiester transmembrane transporter Tgp1 as the importer of the putative toxicity factor. This led us to recapitulate toxicity of the *asp1-STF* mutants by supplementing ePMGT with glycerophosphocholine, which engenders a working model for how increased IP_8_ in *asp1-STF* cells leads to overexpression of *tgp1* and thus increased import of GPC. Induced overexpression of *tgp1* in *asp1*^+^ wild-type cells also elicited toxicity dependent on GPC in the medium. It is conceivable that elevated levels of intracellular GPC are toxic *per se* (e.g., by perturbing phospholipid dynamics) or that elevated GPC engenders its conversion into elevated levels of derivatives that are toxic.

## MATERIALS AND METHODS

### Spot tests of fission yeast growth

Cultures of *S. pombe* strains were grown in liquid ePMGT or YES medium until *A*_600_ reached 0.3–0.8. The cultures were adjusted to an *A*_600_ of 0.1, and aliquots (3 µL) of serial fivefold dilutions were spotted to ePMGT or YES agar. The plates were photographed after incubation for 2 days at 34°C, 2–2.5 days at 30°C and 37°C, 4 days at 25°C, and 6 days at 20°C.

### Acid phosphatase activity

Cells were grown at 30°C in ePMGT or YES medium. Aliquots of exponentially growing cultures were harvested, washed with water, and resuspended in water. To quantify acid phosphatase activity, reaction mixtures (200 µL) containing 100 mM sodium acetate (pH 4.2), 10 mM *p*-nitrophenylphosphate, and cells (ranging from 0.01 to 0.1 *A*_600_ units) were incubated for 5 min at 30°C. The reactions were quenched by addition of 1 mL of 1 M sodium carbonate, the cells were removed by centrifugation, and the absorbance of the supernatant at 410 nm was measured. Acid phosphatase activity is expressed as the ratio of *A*_410_ (*p*-nitrophenol production) to *A*_600_ (cells). The data are averages (±SEM) of at least three assays using cells from three independent cultures.

### *SST* suppressor screen

*STF6::hygMX* and *STF9::hygMX* cells were streaked on ePMG agar, and single colonies were then patched to ePMG agar. Cells from individual patches were suspended in YES medium and then plated at high density to YES + hygromycin agar. Candidate suppressors that formed large colonies after 2–3 days at 30°C were selected, and their growth was assessed in parallel with the respective parent *STF6* or *STF9* strains on YES and ePMG. The suppressor strains were then subjected to whole-genome sequencing.

### Whole-genome sequencing

After PicoGreen quantification and quality control by Agilent BioAnalyzer, 500 ng aliquots of genomic DNA were sheared using a LE220-plus Focused-ultrasonicator (Covaris catalog #500569). Sequencing libraries were prepared using the KAPA Hyper Prep Kit (Kapa Biosystems KK8504) with modifications. DNA libraries were subjected to size selection by mixture with 0.5 vol of aMPure XP beads (Beckman Coulter catalog # A63882) after post-ligation cleanup. Libraries were not amplified by PCR and were pooled equivolume for sequencing. Samples were run on a NovaSeq 6000 in a 150-bp/150-bp paired-end run using the NovaSeq 6000 SBS v1 Kit and an S1 flow cell (Illumina). The average number of read pairs per sample was 10 million.

### Mapping suppressor mutations

The FASTA file for the *S. pombe* genome was accessed from Pombase. The whole-genome sequencing data from the parental *STF6* and *STF9* cells and the suppressor mutants were aligned to the genome using Bowtie2 (31). The resulting SAM files were converted to BAM files using Samtools (32). Variants were identified by BCFtools (33) using the criteria of adjusted mapping quality = 40, minimum base quality = 20, and disabled probabilistic realignment for the computation of base alignment quality for considering variations or insertion-deletion events. The multi-allelic caller protocol was used for variant calling in BCFtools. Variants were annotated using SnpEff, with its in-built genome version for *S. pombe* (34). Variants were further filtered by removing all variations with an average mapping quality ≤25 (Phred scale). All variants present in the parental strain were excluded as noncausal mutations.

### *tgp1* deletion strain

The *tgp1* knockout integration cassette was generated in a bacterial pKS-based plasmid by standard cloning procedures. The integration cassette consisted of the following elements, proceeding from 5′ to 3′: (i) a 540-bp segment of genomic DNA spanning nucleotides −535 to +5 relative to the *nctgp1* transcription start site; (ii) a 795-bp segment of genomic DNA consisting of the *ura4*^+^ ORF; (iii) a 245-bp segment of genomic DNA 3′ of the *Ashbya gossypii TEF* stop codon containing cleavage/polyadenylation and termination signals; and (iv) a 322-bp segment of genomic DNA 3′ of the *tgp1*^+^ stop codon. The linear *tgp1*∆*::ura4*^+^ gene disruption cassette was excised from the pKS plasmid and transfected into uracil auxotrophic diploid *S. pombe* cells. Uracil prototrophic transformants were selected and analyzed by Southern blotting to confirm correct integration at the *tgp1* locus. Confirmed heterozygous diploids were sporulated, and uracil-prototrophic haploid *tgp1*∆ progeny was selected.

### Allelic exchange at the *kcs1* locus

Strains harboring marked wild-type and *SST-kcs1* alleles were constructed as follows. We first generated a pUC18-based plasmid harboring an integration cassette for wild-type *kcs1* that consisted of four elements in series from 5′ to 3′: (i) a 3.68-kb segment of genomic DNA encompassing the *kcs1* ORF plus 775 bp upstream of the *kcs1* start codon (*kcs1* locus −775 to +2904 with +1 being the translation start); (ii) a 282-bp segment including the *ADH1* terminator; (iii) a *kanMX* cassette conferring resistance to G418; and (iv) a 734-bp DNA segment of genomic DNA 3′ of the *kcs1* stop codon. Missense mutations in the *kcs1* ORF were introduced by exchanging wild-type restriction fragments with DNA segments amplified from the respective *SST* suppressor strains. All inserts were sequenced to exclude the presence of unwanted mutations. The integration cassettes were excised and transfected into diploid *S. pombe* cells. Kanamycin-resistant transformants were selected, and correct integrations at the target locus were confirmed by Southern blotting. The heterozygous diploids were then sporulated, and kanamycin-resistant haploids were isolated. A segment of the *kcs1-kanMX* locus was amplified by PCR and sequenced to verify the presence of the respective mutation.

### Transcriptome profiling by RNA-seq

RNA was isolated from *S. pombe* wild-type, *asp1-STF6,* and *asp1-STF9* cells that were grown in liquid ePMGT medium and from wild-type, *kcs1-R332T*, *kcs1-L338R*, and *kcs1-E834K* cells that were grown in liquid YES medium at 30°C to an *A*_600_ of 0.5–0.6. Cells were harvested by centrifugation, and total RNA was extracted via the hot phenol method. The integrity of total RNA was gauged with an Agilent Technologies 2100 Bioanalyzer. The NEB Ultra II Directional RNA library Prep plus Poly A isolation module kit was used to prepare the libraries for paired-end sequencing using a NovaSeq 6000 system. FASTQ files bearing paired-end reads of length 51 bases were mapped to the *S. pombe* genome (ASM294v2.28) using HISAT2-2.1.0 with default parameters (35). The resulting SAM files were converted to BAM files using Samtools (32). Count files for individual replicates were generated with HTSeq-0.10.0 (36) using exon annotations from Pombase (GFF annotations, genome-version ASM294v2; source “ensembl”). RPKM analysis and pairwise correlations (Fig. S4 and S6) were performed as described previously (37). Differential gene expression and fold change analysis were performed in DESeq2 (38). Cutoff for further evaluation was set for genes that had an adjusted *P*-value (Benjamini-Hochberg corrected) of ≤0.05 and were up or down by at least twofold in *STF6*, *STF9*, *kcs1-R332T*, *kcs1-L338R,* or *kcs1-E834K* versus wild-type. Genes were further filtered on the following criteria: (i) genes that were ≥2-fold up and the average normalized read count for the mutant strain was ≥100 and (ii) genes that were ≥2-fold down and the average normalized read count for the wild-type strain was ≥100.

## Data Availability

The RNA-seq data in this publication have been deposited in NCBI's Gene Expression Omnibus and are accessible through GEO Series accession number GSE241189.

## SUPPLEMENTAL MATERIAL

Supplemental material mbio.03062-23-s0001.docx Figures S1 to S6 and Table S3.

Table S1 mbio.03062-23-s0002.pdf Genes that are dysregulated in STF6 and STF9.

Table S2 mbio.03062-23-s0003.pdf Genes that are dysregulated in *kcs1* mutants.

ASM does not own the copyrights to Supplemental Material that may be linked to, or accessed through, an article. The authors have granted ASM a non-exclusive, world-wide license to publish the Supplemental Material files. Please contact the corresponding author directly for reuse.
