# Supplementary material for: Genetic suppressor screen identifies Tgp1 (glycerophosphocholine transporter), Kcs1 (IP6 kinase), and Plc1 (phospholipase C) as determinants of inositol pyrophosphate toxicosis in fission yeast
Source: mBio. 2023 Dec 22;15(2):e03062-23. doi: 10.1128/mbio.03062-23 (PMC10865970; doi:10.1128/mbio.03062-23)
Supplement: Supplemental material — Figures S1 to S6 and Table S3. [file mbio.03062-23-s0001.docx]

Supplemental Material

**Genetic suppressor screen identifies Tgp1 (glycerophosphocholine transporter), Plc1 (phospholipase C), and Kcs1 (IP_6_ kinase) as determinants of inositol pyrophosphate toxicosis in fission yeast**

Lauren Bednor, Ana M. Sanchez, Angad Garg, Stewart Shuman, and Beate Schwer

Supplemental Figures S1, S2, S3, S4, S5, S6

Supplemental Tables S1, S2, S3

[Tables S1 and S2 are separately uploaded as PDFs generated from .xls files]


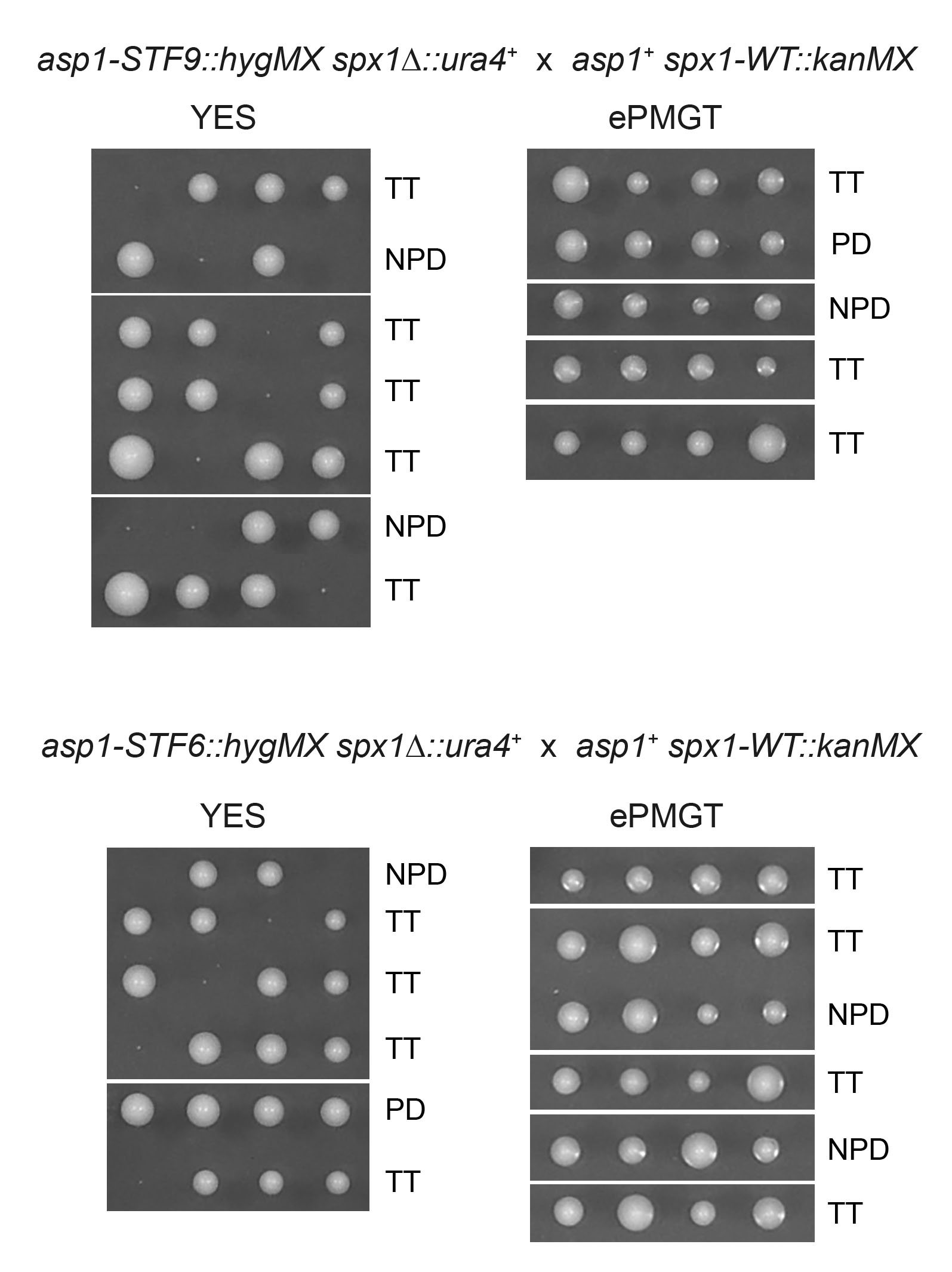


Figure S1. Tetrad dissection on YES and ePMGT agar. Diploids obtained by crossing *asp1-STF9::hygMX* *spx1∆::ura4*^+^ and *STF6::hygMX* *spx1∆::ura4*^+^ strains to *asp1*^+^ *spx1-WT::kanMX* cells were selected on kanamycin and hygromycin-containing agar plates and then subjected to sporulation on malt agar for 24-48 h. Individual spores of tetrads were arrayed on YES or ePMGT agar medium and incubated for 5 d (YES) or 6 d (ePMGT) at 30°C. Genotyping the viable progeny assigned the segregation pattern as tetratype (TT), non-parental ditype (NPD), or parental ditype (PD) as indicated.


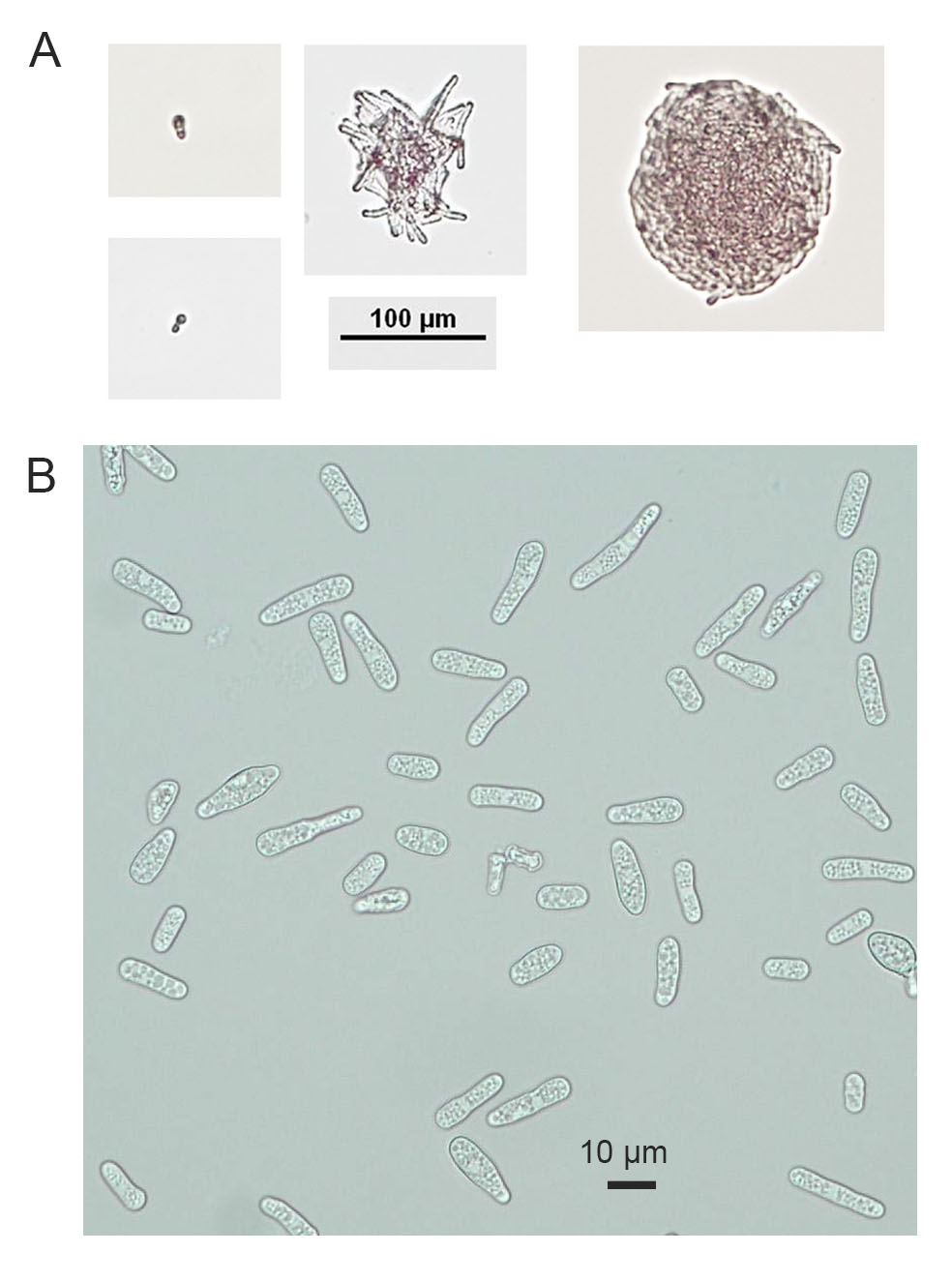


Figure S2. *asp1-STF6 spx1-WT* haploids on YES agar plates. Upon tetrad dissection and incubation of the YES plates at 30°C, individual spores were observed microscopically. (A) Photographs of spores (representative examples) that did not progress to form macroscopic colonies after 5-7 days (and were identified as *asp1-STF6 spx1-WT* haploids upon genotyping of the viable spores of the tetrad). (B) Photograph of *asp1-STF6 spx1-WT* cells from a microcolony (e.g., right-most picture in panel A) that was resuspended in liquid.


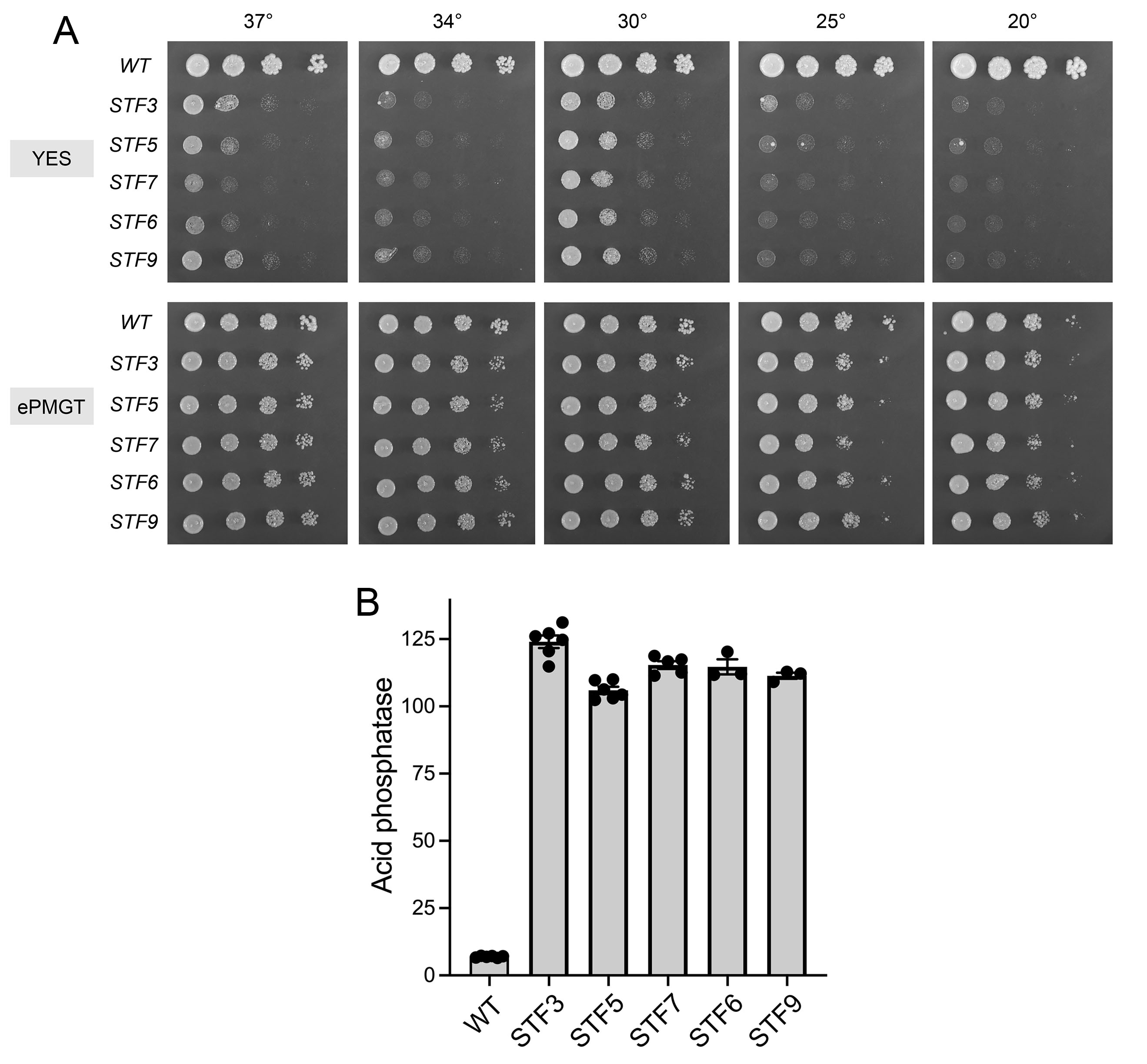


Figure S3. Growth defects of all *asp1-STF* mutants on YES medium are rescued on ePMGT medium. (A) Serial 5-fold dilutions of fission yeast strains (as specified on the left) were spot tested for growth on YES agar and on ePMGT agar at the indicated temperatures. (B) The indicated strains were grown to *A*_600_ of 0.5 to 0.8 in liquid culture in ePMGT medium at 30˚C. Cells were then harvested, washed with water, and assayed for Pho1 acid phosphatase activity by conversion of *p*-nitrophenylphosphate to *p*-nitrophenol. Activity is expressed as the ratio of *A*_410_ (*p*-nitrophenol production) to *A*_600_ (input cells).

| **Sample** | **Total Paired Reads** | **Mapped Reads** |
| --- | --- | --- |
| *WT* (1) | 39,560,485 | 38,485,062 (97%) |
| *WT* (2) | 31,946,110 | 29,876,115 (94%) |
| *WT* (3) | 31,128,247 | 29,356,402 (94%) |
| *STF6* (1) | 31,256,580 | 29,195,047 (93%) |
| *STF6* (2) | 30,814,765 | 29,052,940 (94%) |
| *STF6* (3) | 31,687,008 | 29,839,725 (94%) |
| *STF9* (1) | 34,389,382 | 32,353,696 (94%) |
| *STF9* (2) | 37,810,104 | 35,144,516 (93%) |
| *STF9* (3) | 27,521,749 | 24,634,934 (90%) |

| **Sample pairs** | **Pearson Coefficient** |
| --- | --- |
| *WT* (1) vs (2) | 0.979 |
| *WT* (2) vs (3) | 0.978 |
| *WT* (1) vs (3) | 0.982 |
| *STF6* (1) vs (2) | 0.981 |
| *STF6* (2) vs (3) | 0.980 |
| *STF6* (1) vs (3) | 0.968 |
| *STF9* (1) vs (2) | 0.981 |
| *STF9* (2) vs (3) | 0.983 |
| *STF9* (1) vs (3) | 0.978 |

Figure S4. RNA-seq read counts and data reproducibility for triplicate biological replicates.


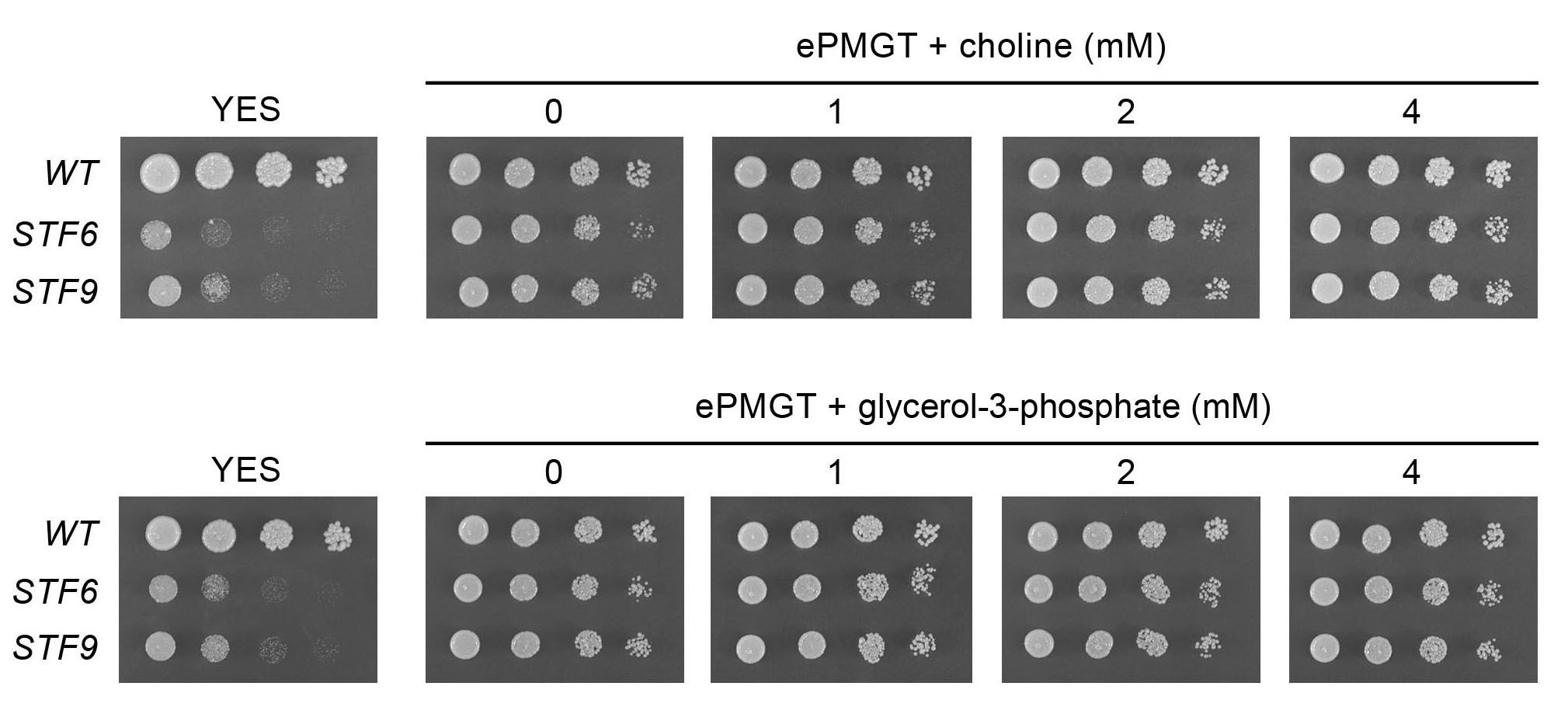


Figure S5. Neither choline nor glycerol-3-phosphate is toxic to *STF6* and *STF9* cells. Serial 5-fold dilutions of the indicated yeast strains were spot-tested for growth at 30˚C on YES agar and on ePMGT agar supplemented with choline (top panel) or glycerol-3-phosphate (bottom panel) as specified.

| **Sample** | **Total Paired Reads** | **Mapped Reads** |
| --- | --- | --- |
| *kcs1-WT* (1) | 27,332,279 | 27,070,472 (99%) |
| *kcs1-WT* (2) | 25,189,585 | 24,955,792 (99%) |
| *kcs1-WT* (3) | 27,719,728 | 27,449,039 (99%) |
| *kcs1-R332T* (1) | 25,724,650 | 25,478,634 (99%) |
| *kcs1-R332T* (2) | 27,478,921 | 27,232,216 (99%) |
| *kcs1-R332T* (3) | 23,471,808 | 23,253,555 (99%) |
| *kcs1-L338R* (1) | 25,818,684 | 25,586,152 (99%) |
| *kcs1-L338R* (2) | 23,654,227 | 23,463,652 (99%) |
| *kcs1-L338R* (3) | 26,314,680 | 26,071,770 (99%) |
| *kcs1-E834K* (1) | 25,690,002 | 25,486,834 (99%) |
| *kcs1-E834K* (2) | 29,709,436 | 29,451,937 (99%) |
| *kcs1-E834K* (3) | 20,928,346 | 20,744,813 (99%) |

| **Sample pairs** | **Pearson Coefficient** |
| --- | --- |
| *kcs1-WT* (1) vs (2) | 0.982 |
| *kcs1-WT* (1) vs (3) | 0.983 |
| *kcs1-WT* (2) vs (3) | 0.982 |
| *kcs1-R332T* (1) vs (2) | 0.985 |
| *kcs1-R332T* (1) vs (3) | 0.984 |
| *kcs1-R332T* (2) vs (3) | 0.983 |
| *kcs1-L338R* (1) vs (2) | 0.970 |
| *kcs1-L338R* (1) vs (3) | 0.979 |
| *kcs1-L338R* (2) vs (3) | 0.981 |
| *kcs1-E834K* (1) vs (2) | 0.984 |
| *kcs1-E834K* (1) vs (3) | 0.983 |
| *kcs1-E834K* (2) vs (3) | 0.983 |

Figure S6. RNA-seq read counts and data reproducibility for triplicate biological replicates.

Table S1. Summary of RNA-seq analysis of *asp1-STF6* and *asp1-STF9* cells.

Table S2. Summary of RNA-seq analysis of *kcs1-R332T*, *kcs1-L338R*, and *kcs1-E834K* cells.

| Strain | Genotype | Source |
| --- | --- | --- |
| JS77 | *h- leu1-32 ura4-D18 his3-D1 ade6-m216* | 1 |
| JS78 | *h+ leu1-32 ura4-D18 his3-D1 ade6-m210* | 1 |
| BS200 | *h- STF3 [asp1-G863D] rpb1::kanMX* | 2 |
| BS202 | *h+ STF5 [asp1C643Y] rpb1::kanMX* | 2 |
| BS204 | *h- STF7 [asp1-H686Y] rpb1::kanMX* | 2 |
| BS216 | *h+ asp1-WT::hygMX* | 2 |
| BS499 | *h+ SST-56 STF5::hygMX [asp1-C643Y] kcs1-S761F* | this study |
| BS500 | *h- SST-77 STF7 [asp1-H686Y] kcs1-L338R* | this study |
| BS848 | *h+ STF6::hygMX [asp1-W386*]* | this study |
| BS858 | *h+ STF9::hygMX [asp1-W493*]* | this study |
| BS849 | *h+ SST-61 STF6::hygMX [asp1-W386*] ssu72*-(-1fs in stop codon) | this study |
| BS850 | *h+ SST-62 STF6::hygMX [asp1-W386*] plc1-A859V* | this study |
| BS854 | *h+ SST-66 STF6::hygMX [asp1-W386*] tgp1-Glu17** | this study |
| BS855 | *h+ SST-67 STF6::hygMX [asp1-W386*] kcs1-R332T pet127* (aa 8 +1fs) | this study |
| BS861 | *h+ SST-93 STF9::hygMX [asp1-W493*] plc1-P708L* | this study |
| AGP237 | *h- tgp1∆::ura4^+^* | this study |
| BS951 | *h- tgp1∆::ura4+ STF6::hygMX [asp1-W386*]* | this study |
| BS940 | *h+ plc1-A859V* | this study |
| BS977 | *h+ plc1-P708L* | this study |
| BS1037 | *h+ kcs1-WT::kanMX* | this study |
| BS1038 | *h+ kcs1-R332T::kanMX* | this study |
| BS1040 | *h+ kcs1-L338R::kanMX* | this study |
| BS1042 | *h+ kcs1-E834K::kanMX* | this study |
| AS2183 | *h+ asp1∆::natMX* | 3 |
| BS476 | *h- spx1-WT::kanMX* | 4 |
| BS1100 | *h+ STF6::hygMX [asp1-W386*] spx1∆::ura4^+^* | this study |
| BS1104 | *h+ STF9::hygMX [asp1-W493*] spx1∆::ura4^+^* | this study |

Table S3. Fission yeast strains used in this study

All strains are derived from the parental strains (JS77 and JS78). The strains are *leu1-32* *ura4-D18* *his3-D1* and either *ade6-m216 or ade6-m210*. The *asp1* alleles in the *STF* mutants are specified in brackets.

1. Pei Y, Du H, Singer J, St Amour C, Granitto S, Shuman S, Fisher RP. (2006) Cyclin-dependent kinase 9 (Cdk9) of fission yeast is activated by the CDK-activating kinase Csk1, overlaps functionally with the TFIIH-associated kinase Mcs6, and associates with the mRNA cap methyltransferase Pcm1 in vivo. *Mol Cell Biol* 26:777-788.
2. Garg A, Shuman S, Schwer B. (2020) A genetic screen for suppressors of hyper-repression of the fission yeast *PHO* regulon by Pol2 CTD mutation T4A implicates inositol 1-pyrophosphates as agonists of precocious lncRNA transcription termination. *Nucleic Acids Res* 48:10739-10752.
3. Sanchez AM, Garg A, Shuman S, Schwer B. (2019) Inositol pyrophosphates impact phosphate homeostasis via modulation of RNA 3’ processing and transcription termination. *Nucleic Acids Res* 47: 8452-8469.
4. Schwer B, Garg A, Sanchez AM, Bernstein MA, Benjamin B, Shuman S. (2022) Cleavage-polyadenylation factor Cft1 and SPX domain proteins are agents of inositol pyrophosphate toxicosis in fission yeast. *mBio* 13: e0347621.
