# Supplementary material for: Genetic suppressor screen identifies Tgp1 (glycerophosphocholine transporter), Kcs1 (IP6 kinase), and Plc1 (phospholipase C) as determinants of inositol pyrophosphate toxicosis in fission yeast
Source: mBio. 2023 Dec 22;15(2):e03062-23. doi: 10.1128/mbio.03062-23 (PMC10865970; doi:10.1128/mbio.03062-23)
Supplement: Table S1 — Genes that are dysregulated in STF6 and STF9. [file mbio.03062-23-s0002.pdf]

SystematicID	Gene.name	Product	STF6_log2FoldChange
SPBC1271.09	tgp1	plasma membrane glycerophosphodiester transmembrane transporter	5.42
SPBPB2B2.06c		extracellular 5'-nucleotidase, human NT5E family	4.05
SPBC8E4.12c	ecl3	extender of chronological lifespan protein Ecl3	3.69
SPBP4G3.02	pho1	extracellular acid phosphatase Pho1	3.40
SPAPJ695.02		Schizosaccharomyces pombe specific protein	3.22
SPBC354.12	gpd3	glyceraldehyde 3-phosphate dehydrogenase Gpd3	2.90
SPBPB2B2.05		class I glutamine amidotransferase family protein	2.80
SPBC56F2.06	mug147	Schizosaccharomyces specific protein Mug147	2.61
SPCC188.12	spn6	meiotic (sporulation) septin Spn6	2.51
SPAPB8E5.10	min8	mitochondrial mini protein, single membrane pass Min8	2.46
SPCC1281.04	akr7	pyridoxal reductase	2.46
SPAC977.16c	dak2	dihydroxyacetone kinase Dak2	2.41
SPAC15E1.02c		DUF1761 family protein	2.25
SPBC8E4.01c	pho84	plasma membrane inorganic phosphate transmembrane transporter	2.22
SPBC725.10	tps0	mitochondrial outer membrane protein, implicated in lipid/sterol transport	2.07
SPBC19C7.04c		DUF2406 family conserved fungal protein	2.06
SPBC1289.16c	cao2	copper amine oxidase-like protein Cao2	1.97
SPAC23D3.12	pho842	plasma membrane inorganic phosphate transmembrane transporter Pho842	1.89
SPBC725.06c	ppk31	serine/threonine protein kinase Ppk31	1.89
SPAC17A2.11		dubious	1.87
SPAPJ691.02		yippee-like protein	1.77
SPAC1F8.04c		hydrolase, implicated in cellular detoxification	1.77
SPAC31G5.09c	spk1	MAP kinase Spk1	1.73
SPBC1347.12	arp1	dynactin complex subunit, centractin family actin-like protein Arp1	1.73
SPBC1861.01c	cnp3	CENP-C ortholog Cnp3	1.69
SPAC11E3.06	map1	DNA-binding transcription factor, MADS-box Map1	1.69
SPAC22F3.12c	rgs1	regulator of G-protein signaling Rgs1	1.66
SPAC27D7.03c	mei2	RNA-binding protein involved in meiosis Mei2	1.59
SPAPB15E9.02c		dubious	1.59
SPAC13F5.07c	hpz2	zf PARP type zinc finger protein Hpz2	1.55
SPBC216.02	num1	meiotic cortical anchoring factor for dynein Mcp5/Num1	1.54
SPAC17A5.18c	rec25	meiotic recombination protein Rec25	1.54
SPAC3F10.10c	map3	pheromone M-factor receptor Map3	1.51
SPAC24C9.15c	spn5	meiotic septin Spn5	1.46
SPBC1652.01	stb3	DNA-binding transcription factor Stb3	1.44
SPCC338.12	pbi2	vaculoar proteinase B inhibitor Pbi2	1.43
SPBC1861.02	abp2	unknown protein, may bind replication origins Abp2	1.42
SPCC417.16		mitochondrial protein	1.40
SPAC139.05		succinate-semialdehyde dehydrogenase	1.38
SPBC32C12.02	ste11	DNA-binding transcription factor Ste11	1.36
SPAC56F8.15		Schizosaccharomyces pombe specific protein	1.33
SPAC22A12.06c	fsh2	serine hydrolase-like, human TSTD2 and OVCA2 ortholog	1.30
SPAC29A4.11	rga3	RhoGAP, GTPase activating protein Rga3	1.30
SPAC1565.04c	ste4	MAPK cascade adaptor protein Ste4	1.29
SPBC317.01	mbx2	DNA-binding transcription factor, MADS-box Pvg4	1.29
SPAC3C7.14c	obr1	NAD(P)H dehydrogenase (quinone)	1.28
SPCC4E9.01c	rec11	meiotic cohesin complex STAG protein Rec11	1.28
SPAC4H3.03c		glucan 1,4-alpha-glucosidase	1.27
SPAPYUG7.06	sdu1	PPPDE peptidase family deubiquitinase/desumoylase Sdu1	1.27
SPBC530.11c		DNA-binding transcription factor, zf-fungal binuclear cluster type	1.27
SPCC23B6.05c	ssb3	DNA replication factor A subunit Ssb3	1.27
SPAC1556.06	meu1	Schizosaccharomyces specific protein	1.26
SPCC330.06c	pmp20	thioredoxin-related chaperone Pmp20	1.24
SPAC29B12.12	hot13	helper of TIM Hot13	1.24
SPBC3B8.08		Sjogren's syndrome/scleroderma autoantigen 1 family	1.23
SPBC1685.17		Schizosaccharomyces pombe specific protein	1.21
SPCC330.04c	mug135	DUF1773 family protein, with repeat expansion	1.20
SPCC1393.14	ten1	nuclear telomere cap complex subunit Ten1	1.15
SPAC5H10.01	dgc1	mitochondrial D-glutamate cyclase Dgc1	1.14
SPBC21B10.04c	nrf1	vacuolar transporter chaperone (VTC) complex, GTPase regulator subunit	1.13
SPAC17A2.07c		Schizosaccharomyces specific protein	1.11
SPBC947.04	pfl3	cell surface glycoprotein, flocculin Pfl3, DIPSY family	1.10
SPAC637.03		DUF1774 family multi-spanning conserved fungal membrane protein	1.10
SPAP8A3.04c	hsp9	heat shock protein Hsp9	1.08
SPAC32A11.01	mug8	DUF1708 family conserved fungal protein, cell division site	1.05
SPCC965.07c	gst2	glutathione S-transferase Gst2	1.04
SPBC1683.01	pho841	plasma membrane inorganic phosphate transmembrane transporter Pho841	1.03
SPBC1198.14c	fbp1	fructose-1,6-bisphosphatase Fbp1	1.01
SPAPB8E5.04c	npc2	Niemann-Pick disease type C2 protein hE1 homolog Npc2	1.01

SystematicID	Gene.name	Product	STF6_log2FoldChange
SPCC569.01c		DUF1773 family protein	-5.62
SPBPB10D8.03		pseudogene transporter	-5.17
SPAC977.18		conserved fungal plasma membrane protein	-4.14
SPCC569.03		DUF1773 family protein	-3.91
SPAC750.01		NADP-dependent aldo/keto reductase	-3.77
SPCP20C8.02c		S. pombe specific UPF0321 family protein 1	-3.59
SPBC359.02	alr2	alanine racemase Alr2	-3.48
SPBPB10D8.02c		arylsulfatase	-3.05
SPBC359.04c	pfl7	cell surface glycoprotein, flocculin Pfl7, DIPSY family	-2.71
SPCC1235.14	ght5	plasma membrane high-affinity glucose/fructose:proton symporter	-2.61
SPBPB2B2.09c	pan5	2-dehydropantoate 2-reductase Pan5	-2.57
SPBC1683.06c	urh1	uridine ribohydrolase Urh1	-2.21
SPBC359.01		amino acid transmembrane transporter	-2.19
SPCC70.12c	ecl1	extender of chronological lifespan protein Ecl1	-2.19
SPBC1271.10c		transmembrane transporter	-2.18
SPBPB21E7.07	aes1	phenazine biosynthesis PhzF protein family	-2.16
SPBC8E4.03		agmatinase 2	-2.16
SPAC922.07c	atd2	aldehyde dehydrogenase	-2.15
SPCC70.08c		methyltransferase	-2.14
SPCC548.07c	ght1	plasma membrane high-affinity glucose:proton symporter	-2.10
SPAC11D3.09		agmatinase	-2.01
SPBC359.03c	aat1	plasma membrane amino acid transmembrane transporter	-2.01
SPAPB24D3.03		agmatinase	-2.00
SPCC1235.17		dubious	-1.98
SPAC11D3.01c		Con-6 family conserved fungal protein	-1.96
SPCC1235.18		dubious	-1.93
SPBC947.05c	frp2	plasma membrane ferric-chelate reductase Frp2	-1.93
SPAC1A6.04c	plb1	phospholipase B homolog Plb1	-1.87
SPBPB21E7.04c	cmt2	O-methyltransferase, human COMT catechol homolog 2	-1.86
SPAC1002.19	urg1	GTP cyclohydrolase II Urg1	-1.86
SPAC11D3.06		MatE family transmembrane transporter	-1.83
SPBC1683.08	ght4	plasma membrane hexose:proton symporter	-1.81
SPAC1002.17c	urg2	uracil phosphoribosyltransferase	-1.76
SPBC1271.07c		N-acetyltransferase	-1.72
SPAC977.15		dienelactone hydrolase family, implicated in cellular detoxification	-1.70
SPBC359.05	abc3	vacuolar heme ABC transmembrane exporter Abc3	-1.61
SPBPB10D8.01		cysteine transmembrane transporter	-1.57
SPAC1002.18	urg3	DUF1688 family fungal conserved protein	-1.48
SPAC1399.04c	uck2	uracil phosphoribosyltransferase Uck2	-1.48
SPCC1884.01		Schizosaccharomyces pombe specific protein	-1.44
SPAC13G7.12c	eki1	choline/ethanolamine kinase Eki1	-1.44
SPBC32H8.02c	nep2	NEDD8 protease Nep2	-1.42
SPCC1682.08c	mpf2	meiotic pumilio family RNA-binding protein Mpf2	-1.42
SPBC1683.09c	frp1	plasma membrane ferric-chelate reductase Frp1	-1.41
SPAC521.03		short chain dehydrogenase, human DHRS7 family	-1.40
SPAC11D3.03c		aminomethyltransferase-like and DUF1989 family protein	-1.39
SPBC1271.08c		Schizosaccharomyces pombe specific protein	-1.38
SPCC584.16c		Schizosaccharomyces specific protein	-1.37
SPAC10F6.07c	mug94	Schizosaccharomyces pombe specific protein Mug94	-1.35
SPAC56E4.03		aromatic aminotransferase	-1.29
SPBP26C9.02c	car1	arginase Car1	-1.29
SPCC794.03		amino acid transmembrane transporter	-1.29
SPBC25B2.11	pof2	F-box protein Pof2	-1.29
SPCC70.03c	put1	proline dehydrogenase Put1	-1.28
SPCC576.17c		pyridoxamine/pyridoxine/pyridoxal transmembrane transporter	-1.28
SPAC19E9.03	pas1	cyclin Pas1	-1.20
SPCC16A11.01	sfk1	plasma membrane protein involved in inositol lipid-mediated signaling	-1.19
SPAC25B8.13c	isp7	2-OG-Fe(II) oxygenase superfamily protein	-1.17
SPBC409.11	meu18	Schizosaccharomyces specific protein Meu18	-1.16
SPCC569.07		aromatic aminotransferase	-1.16
SPCC1281.08	wtf11	wtf element Wtf11	-1.16
SPAC5H10.10		NADPH dehydrogenase, (Old yellow enzyme)	-1.12
SPCC191.06		Schizosaccharomyces pombe specific protein	-1.12
SPAC869.10c	put4	plasma membrane proline transmembrane transporter Put4	-1.12
SPAC10F6.01c	sir1	sulfite reductase beta subunit Sir1	-1.11
SPAC1B3.16c	vht1	plasma membrane vitamin H transmembrane transporter Vht1	-1.09
SPBC1773.17c	gor2	glyoxylate reductase	-1.09
SPBPB21E7.08		pseudogene	-1.08
SPCC965.14c		cytosine deaminase	-1.08
SPAP7G5.06	per1	plasma membrane amino acid transmembrane transporter Per1	-1.08
SPCC364.07	ser3	D-3 phosphoglycerate dehydrogenase Ser3	-1.07
SPAC11D3.17		DNA-binding transcription factor, zf-fungal binuclear cluster type	-1.05
SPCC31H12.05c	sds21	serine/threonine protein phosphatase PP1 catalytic subunit Sds21	-1.05
SPBC1773.14	arg7	argininosuccinate lyase	-1.05
SPAC21E11.04	aca1	L-azetidine-2-carboxylic acid acetyltransferase Aca1	-1.04
SPAC11D3.02c		ELLA family acetyltransferase	-1.03
SPCC18B5.01c	bfr1	plasma membrane brefeldin A efflux transporter Bfr1	-1.03
SPAC1399.05c	toe1	DNA-binding transcription factor, zf-fungal binuclear cluster type	-1.03
SPCC191.11	inv1	external invertase, beta-fructofuranosidase Inv1	-1.02
SPAC16E8.16	sua7	transcription factor TFIIB	-1.01

SystematicID	Gene.name	Product	STF9_log2FoldChange
SPBC1271.09	tgp1	plasma membrane glycerophosphodiester transmembrane transporter	5.55
SPBPB2B2.06c		extracellular 5'-nucleotidase, human NT5E family	4.19
SPBC8E4.12c	ecl3	extender of chronological lifespan protein Ecl3	3.71
SPBP4G3.02	pho1	extracellular acid phosphatase Pho1	3.63
SPBC1289.14		adducin	3.20
SPBC354.12	gpd3	glyceraldehyde 3-phosphate dehydrogenase Gpd3	3.06
SPAPJ695.02		Schizosaccharomyces pombe specific protein	2.86
SPAC977.16c	dak2	dihydroxyacetone kinase Dak2	2.77
SPAC15E1.02c		DUF1761 family protein	2.58
SPBPB2B2.05		class I glutamine amidotransferase family protein	2.56
SPBC725.10	tps0	mitochondrial outer membrane protein	2.52
SPBC56F2.06	mug147	Schizosaccharomyces specific protein Mug147	2.45
SPBC8E4.01c	pho84	plasma membrane inorganic phosphate transmembrane transporter	2.43
SPCC1281.04	akr7	pyridoxal reductase	2.38
SPBC19C7.04c		DUF2406 family conserved fungal protein	2.16
SPAC31G5.09c	spk1	MAP kinase Spk1	2.13
SPCC188.12	spn6	meiotic (sporulation) septin Spn6	2.12
SPAC23D3.12	pho842	plasma membrane inorganic phosphate transmembrane transporter Pho842	2.04
SPAC17A2.11		dubious	1.97
SPAPJ691.02		yippee-like protein	1.91
SPAC3F10.10c	map3	pheromone M-factor receptor Map3	1.88
SPAC27D7.03c	mei2	RNA-binding protein involved in meiosis Mei2	1.81
SPAC22F3.12c	rgs1	regulator of G-protein signaling Rgs1	1.81
SPAC56F8.15		Schizosaccharomyces pombe specific protein	1.80
SPBC1289.16c	cao2	copper amine oxidase-like protein Cao2	1.77
SPAPB15E9.02c		dubious	1.76
SPBC1347.12	arp1	dynactin complex subunit, centractin family actin-like protein Arp1	1.70
SPBC317.01	mbx2	DNA-binding transcription factor, MADS-box Pvg4	1.69
SPBC32C12.02	ste11	DNA-binding transcription factor Ste11	1.66
SPAC3C7.14c	obr1	NAD(P)H dehydrogenase (quinone)	1.64
SPCC417.16		mitochondrial protein	1.62
SPBC1861.02	abp2	unknown protein, may bind replication origins Abp2	1.58
SPCC338.12	pbi2	vaculoar proteinase B inhibitor Pbi2	1.58
SPAC11E3.06	map1	DNA-binding transcription factor, MADS-box Map1	1.56
SPBC1652.01	stb3	DNA-binding transcription factor Stb3	1.55
SPAPYUG7.06	sdu1	PPPDE peptidase family deubiquitinase/desumoylase Sdu1	1.53
SPAP8A3.04c	hsp9	heat shock protein Hsp9	1.46
SPBC1861.01c	cnp3	CENP-C ortholog Cnp3	1.44
SPAC4H3.03c		glucan 1,4-alpha-glucosidase	1.44
SPCC330.06c	pmp20	thioredoxin-related chaperone Pmp20	1.42
SPBC21B10.04c	nrf1	vacuolar transporter chaperone (VTC) complex, GTPase regulator subunit	1.41
SPBC530.11c		DNA-binding transcription factor, zf-fungal binuclear cluster type	1.39
SPBPB2B2.01		amino acid transmembrane transporter	1.38
SPAC22A12.06c	fsh2	serine hydrolase-like, human TSTD2 and OVCA2 ortholog,	1.37
SPAC139.05		succinate-semialdehyde dehydrogenase	1.34
SPAC637.03		DUF1774 family multi-spanning conserved fungal membrane protein	1.34
SPAC29B12.12	hot13	helper of TIM Hot13	1.32
SPBC947.04	pfl3	cell surface glycoprotein, flocculin Pfl3, DIPSY family	1.30
SPAC29A4.11	rga3	RhoGAP, GTPase activating protein Rga3	1.28
SPAC9E9.11	plr1	pyridoxal reductase Plr1	1.27
SPAC6C3.02c	mix17	mitochondrial CHCH domain protein Mix17	1.25
SPCC23B6.05c	ssb3	DNA replication factor A subunit Ssb3	1.25
SPAC1F8.04c		hydrolase, implicated in cellular detoxification	1.25
SPBC725.06c	ppk31	serine/threonine protein kinase Ppk31	1.23
SPAPB8E5.04c	npc2	Niemann-Pick disease type C2 protein hE1 homolog Npc2	1.23
SPAC186.01	pfl9	cell surface glycoprotein, flocculin Pfl9, DIPSY family	1.21
SPBC16E9.16c	lsd90	Lsd90 protein	1.21
SPAC32A11.01	mug8	DUF1708 family conserved fungal protein, cell division site	1.19
SPCC162.10	ppk33	serine/threonine protein kinase Ppk33	1.17
SPCC1020.06c	tal1	transaldolase Tal1	1.17
SPBC16A3.13	meu7	alpha-amylase homolog Aah4	1.14
SPBC21C3.10c	rib7	5-amino-6-(5-phosphoribosylamino) uracil reductase Rib7	1.14
SPCC965.07c	gst2	glutathione S-transferase Gst2	1.12
SPAC20H4.11c	rho5	Rho family GTPase Rho5	1.12
SPBC1683.01	pho841	plasma membrane inorganic phosphate transmembrane transporter Pho841	1.11
SPAC13F5.07c	hpz2	zf PARP type zinc finger protein Hpz2	1.07
SPAC1486.01	sod2	mitochondrial superoxide dismutase Sod2	1.07
SPAPB24D3.07c		Schizosaccharomyces pombe specific protein	1.06
SPCC1393.10	ctr4	plasma membrane copper transporter complex subunit Ctr4	1.06
SPAC27D7.09c		But2 family protein, similar to cell surface molecules	1.06
SPCC1795.06	map2	P-factor pheromone Map2	1.05
SPBC83.13	yhm2	mitochondrial carrier, tricarboxylic acid Yhm2	1.05
SPCC622.08c	hta1	histone H2A alpha	1.04
SPBC1703.13c		mitochondrial carrier, inorganic phosphate/copper	1.04
SPAC144.04c	spe1	ornithine decarboxylase Spe1	1.03
SPAC19G12.06c	hta2	histone H2A beta	1.03
SPACUNK4.10	gor1	glyoxylate reductase	1.03
SPAC3G9.11c	pdc201	pyruvate decarboxylase	1.02
SPBC1198.14c	fbp1	fructose-1,6-bisphosphatase Fbp1	1.02
SPBC24C6.09c		phosphoketolase family protein	1.02
SPCC1322.14c	vtc4	vacuolar transporter chaperone (VTC) complex subunit	1.02
SPCC645.03c	isa1	mitochondrial [4Fe-4S] cluster assembly and transfer protein Isa1	1.02
SPBC839.15c	tef103	translation elongation factor EF-1 alpha Ef1a-c	1.01

SystematicID	Gene.name	Product	STF9_log2FoldChange
SPAC750.01		NADP-dependent aldo/keto reductase	-4.47
SPBPB10D8.03		pseudogene transporter	-4.13
SPCP20C8.02c		S. pombe specific UPF0321 family protein 1	-3.71
SPBPB2B2.09c	pan5	2-dehydropantoate 2-reductase Pan5	-2.81
SPBC359.02	alr2	alanine racemase Alr2	-2.75
SPAC11D3.01c		Con-6 family conserved fungal protein	-2.39
SPBPB10D8.02c		arylsulfatase	-2.31
SPCC1235.14	ght5	plasma membrane high-affinity glucose/fructose:proton symporter	-2.22
SPBC1683.06c	urh1	uridine ribohydrolase Urh1	-2.21
SPAC922.07c	atd2	aldehyde dehydrogenase	-2.16
SPBPB21E7.07	aes1	phenazine biosynthesis PhzF protein family	-2.05
SPBC1271.10c		transmembrane transporter	-2.04
SPCC70.12c	ecl1	extender of chronological lifespan protein Ecl1	-1.94
SPCC1884.01		Schizosaccharomyces pombe specific protein	-1.87
SPBC359.04c	pfl7	cell surface glycoprotein, flocculin Pfl7, DIPSY family	-1.82
SPCC1235.18		dubious	-1.81
SPCC70.08c		methyltransferase	-1.79
SPAC977.18		conserved fungal plasma membrane protein	-1.78
SPAC11D3.03c		aminomethyltransferase-like and DUF1989 family protein	-1.77
SPBC8E4.03		agmatinase 2	-1.76
SPBPB21E7.04c	cmt2	O-methyltransferase, human COMT catechol homolog 2	-1.75
SPCC548.07c	ght1	plasma membrane high-affinity glucose:proton symporter Ght1	-1.71
SPBC359.01		amino acid transmembrane transporter	-1.70
SPAC10F6.07c	mug94	Schizosaccharomyces pombe specific protein Mug94	-1.66
SPAC11D3.09		agmatinase	-1.64
SPCC1235.17		dubious	-1.63
SPBC1271.07c		N-acetyltransferase	-1.62
SPAC1002.19	urg1	GTP cyclohydrolase II Urg1	-1.57
SPAC1002.17c	urg2	uracil phosphoribosyltransferase	-1.55
SPAPB24D3.03		agmatinase	-1.55
SPAC1A6.04c	plb1	phospholipase B homolog Plb1	-1.50
SPAC977.15		dienelactone hydrolase family, implicated in cellular detoxification	-1.47
SPBC947.05c	frp2	plasma membrane ferric-chelate reductase Frp2	-1.47
SPBC1683.08	ght4	plasma membrane hexose:proton symporter, unknown specificity Ght4	-1.47
SPBC359.03c	aat1	plasma membrane amino acid transmembrane transporter Aat1	-1.44
SPAC11D3.06		MatE family transmembrane transporter	-1.42
SPCC569.07		aromatic aminotransferase	-1.32
SPCC1682.08c	mpf2	meiotic pumilio family RNA-binding protein Mpf2	-1.29
SPBC359.05	abc3	vacuolar heme ABC transmembrane exporter Abc3	-1.28
SPAC1556.04c	cdd1	cytidine deaminase Ccd1	-1.27
SPCC965.14c		cytosine deaminase	-1.27
SPAC1399.04c	uck2	uracil phosphoribosyltransferase Uck2	-1.27
SPBC1683.02		adenine deaminase	-1.24
SPAC1002.18	urg3	DUF1688 family fungal conserved protein	-1.24
SPAC13G7.12c	eki1	choline/ethanolamine kinase Eki1	-1.22
SPCC584.16c		Schizosaccharomyces specific protein	-1.22
SPBC25B2.11	pof2	F-box protein Pof2	-1.20
SPBC1773.17c	gor2	glyoxylate reductase	-1.17
SPCP20C8.01c		DUF1773 family protein	-1.16
SPBC32H8.02c	nep2	NEDD8 protease Nep2	-1.16
SPAC27D7.08c	mtl16	23S rRNA/U6 snRNA (adenine-N(6))-methyltransferase Mtl16	-1.15
SPBPB21E7.08		pseudogene	-1.15
SPCC16A11.01	sfk1	plasma membrane protein involved in inositol lipid-mediated signaling	-1.14
SPAC11D3.02c		ELLA family acetyltransferase	-1.11
SPAC15A10.10	mde6	Muskelin homolog, kelch repeat, expressed during meiotic cell cycle	-1.11
SPBC1683.09c	frp1	plasma membrane ferric-chelate reductase Frp1	-1.06
SPBC409.11	meu18	Schizosaccharomyces specific protein Meu18	-1.06
SPAC521.03		short chain dehydrogenase, human DHRS7 family	-1.06
SPBC609.02	ptn1	phosphatidylinositol-3,4,5-trisphosphate3-phosphatase Ptn1	-1.05
SPAC25B8.13c	isp7	2-OG-Fe(II) oxygenase superfamily protein	-1.05
SPBC14C8.19	tam10	nucleolar RNA binding protein, human KNOP1 ortholog	-1.04
SPBC1709.06	dus2	tRNA/mRNA dihydrouridine synthase Dus2	-1.04
SPCC794.03		amino acid transmembrane transporter	-1.04
SPBC660.14	mik1	M phase inhibitor protein kinase Mik1	-1.02
SPBC1773.14	arg7	argininosuccinate lyase	-1.02
SPAC26F1.02	pnn1	splicing factor, pinin ortholog Pnn1	-1.02
SPBC1271.08c		Schizosaccharomyces pombe specific protein	-1.01
SPAC56E4.03		aromatic aminotransferase	-1.00
SPCC663.11	saf1	splicing associated factor Saf1	-1.00

SystematicID	Gene.name	Product	STF6_log2FoldChange	STF9_log2FoldChange
SPBC1271.09	tgp1	plasma membrane glycerophosphodiester transmembrane transporter	5.42	5.55
SPBPB2B2.06c		extracellular 5'-nucleotidase, human NT5E family	4.05	4.19
SPBC8E4.12c	ecl3	extender of chronological lifespan protein Ecl3	3.69	3.71
SPBP4G3.02	pho1	extracellular acid phosphatase Pho1	3.40	3.63
SPAPJ695.02		Schizosaccharomyces pombe specific protein	3.22	2.86
SPBC354.12	gpd3	glyceraldehyde 3-phosphate dehydrogenase Gpd3	2.90	3.06
SPBPB2B2.05		class I glutamine amidotransferase family protein	2.80	2.56
SPBC56F2.06	mug147	Schizosaccharomyces specific protein Mug147	2.61	2.45
SPCC188.12	spn6	meiotic (sporulation) septin Spn6	2.51	2.12
SPCC1281.04	akr7	pyridoxal reductase	2.46	2.38
SPAC977.16c	dak2	dihydroxyacetone kinase Dak2	2.41	2.77
SPAC15E1.02c		DUF1761 family protein	2.25	2.58
SPBC8E4.01c	pho84	plasma membrane inorganic phosphate transmembrane transporter	2.22	2.43
SPBC725.10	tps0	mitochondrial outer membrane protein, TspO/MBR-related	2.07	2.52
SPBC19C7.04c		DUF2406 family conserved fungal protein	2.06	2.16
SPBC1289.16c	cao2	copper amine oxidase-like protein Cao2	1.97	1.77
SPAC23D3.12	pho842	plasma membrane inorganic phosphate transmembrane transporter Pho842	1.89	2.04
SPBC725.06c	ppk31	serine/threonine protein kinase Ppk31	1.89	1.23
SPAC17A2.11		dubious	1.87	1.97
SPAPJ691.02		yippee-like protein	1.77	1.91
SPAC1F8.04c		hydrolase, implicated in cellular detoxification	1.77	1.25
SPAC31G5.09c	spk1	MAP kinase Spk1	1.73	2.13
SPBC1347.12	arp1	dynactin complex subunit, centractin family actin-like protein Arp1	1.73	1.70
SPBC1861.01c	cnp3	CENP-C ortholog Cnp3	1.69	1.44
SPAC11E3.06	map1	DNA-binding transcription factor, MADS-box Map1	1.69	1.56
SPAC22F3.12c	rgs1	regulator of G-protein signaling Rgs1	1.66	1.81
SPAC27D7.03c	mei2	RNA-binding protein involved in meiosis Mei2	1.59	1.81
SPAPB15E9.02c		dubious	1.59	1.76
SPAC13F5.07c	hpz2	zf PARP type zinc finger protein Hpz2	1.55	1.07
SPAC3F10.10c	map3	pheromone M-factor receptor Map3	1.51	1.88
SPBC1652.01	stb3	DNA-binding transcription factor Stb3	1.44	1.55
SPCC338.12	pbi2	vaculoar proteinase B inhibitor Pbi2	1.43	1.58
SPBC1861.02	abp2	unknown protein, may bind replication origins Abp2	1.42	1.58
SPCC417.16		mitochondrial protein	1.40	1.62
SPAC139.05		succinate-semialdehyde dehydrogenase	1.38	1.34
SPBC32C12.02	ste11	DNA-binding transcription factor Ste11	1.36	1.66
SPAC56F8.15		Schizosaccharomyces pombe specific protein	1.33	1.80
SPAC22A12.06c	fsh2	serine hydrolase-like, human TSTD2 and OVCA2 ortholog	1.30	1.37
SPAC29A4.11	rga3	RhoGAP, GTPase activating protein Rga3	1.30	1.28
SPBC317.01	mbx2	DNA-binding transcription factor, MADS-box Pvg4	1.29	1.69
SPAC3C7.14c	obr1	NAD(P)H dehydrogenase (quinone)	1.28	1.64
SPAC4H3.03c		glucan 1,4-alpha-glucosidase	1.27	1.44
SPAPYUG7.06	sdu1	PPPDE peptidase family deubiquitinase/desumoylase Sdu1	1.27	1.53
SPBC530.11c		DNA-binding transcription factor, zf-fungal binuclear cluster type	1.27	1.39
SPCC23B6.05c	ssb3	DNA replication factor A subunit Ssb3	1.27	1.25
SPCC330.06c	pmp20	thioredoxin-related chaperone Pmp20	1.24	1.42
SPAC29B12.12	hot13	helper of TIM Hot13	1.24	1.32
SPBC21B10.04c	nrf1	vacuolar transporter chaperone (VTC) complex, GTPase regulator subunit	1.13	1.41
SPBC947.04	pfl3	cell surface glycoprotein, flocculin Pfl3, DIPSY family	1.10	1.30
SPAC637.03		DUF1774 family multi-spanning conserved fungal membrane protein	1.10	1.34
SPAP8A3.04c	hsp9	heat shock protein Hsp9	1.08	1.46
SPAC32A11.01	mug8	DUF1708 family conserved fungal protein, cell division site	1.05	1.19
SPCC965.07c	gst2	glutathione S-transferase Gst2	1.04	1.12
SPBC1683.01	pho841	plasma membrane inorganic phosphate transmembrane transporter Pho841	1.03	1.11
SPBC1198.14c	fbp1	fructose-1,6-bisphosphatase Fbp1	1.01	1.02
SPAPB8E5.04c	npc2	Niemann-Pick disease type C2 protein hE1 homolog Npc2	1.01	1.23

SystematicID	Gene.name	Product	STF6_log2FoldChange	STF9_log2FoldChange
SPBPB10D8.03		pseudogene transporter	-5.17	-4.13
SPAC977.18		conserved fungal protein	-4.14	-1.78
SPAC750.01		NADP-dependent aldo/keto reductase	-3.77	-4.47
SPCP20C8.02c		S. pombe specific UPF0321 family protein 1	-3.59	-3.71
SPBC359.02	alr2	alanine racemase Alr2	-3.48	-2.75
SPBPB10D8.02c		arylsulfatase	-3.05	-2.31
SPBC359.04c	pfl7	cell surface glycoprotein, flocculin Pfl7, DIPSY family	-2.71	-1.82
SPCC1235.14	ght5	plasma membrane high-affinity glucose/fructose:proton symporter	-2.61	-2.22
SPBPB2B2.09c	pan5	2-dehydropantoate 2-reductase Pan5	-2.57	-2.81
SPBC1683.06c	urh1	uridine ribohydrolase Urh1	-2.21	-2.21
SPBC359.01		amino acid transmembrane transporter	-2.19	-1.70
SPCC70.12c	ecl1	extender of chronological lifespan protein Ecl1	-2.19	-1.94
SPBC1271.10c		transmembrane transporter	-2.18	-2.04
SPBPB21E7.07	aes1	phenazine biosynthesis PhzF protein family	-2.16	-2.05
SPBC8E4.03		agmatinase 2	-2.16	-1.76
SPAC922.07c	atd2	aldehyde dehydrogenase	-2.15	-2.16
SPCC70.08c		methyltransferase	-2.14	-1.79
SPCC548.07c	ght1	plasma membrane high-affinity glucose:proton symporter	-2.10	-1.71
SPAC11D3.09		agmatinase	-2.01	-1.64
SPBC359.03c	aat1	plasma membrane amino acid transmembrane transporter	-2.01	-1.44
SPAPB24D3.03		agmatinase	-2.00	-1.55
SPCC1235.17		dubious	-1.98	-1.63
SPAC11D3.01c		Con-6 family conserved fungal protein	-1.96	-2.39
SPCC1235.18		dubious	-1.93	-1.81
SPBC947.05c	frp2	plasma membrane ferric-chelate reductase Frp2	-1.93	-1.47
SPAC1A6.04c	plb1	phospholipase B homolog Plb1	-1.87	-1.50
SPBPB21E7.04c	cmt2	O-methyltransferase, human COMT catechol homolog 2	-1.86	-1.75
SPAC1002.19	urg1	GTP cyclohydrolase II Urg1	-1.86	-1.57
SPAC11D3.06		MatE family transmembrane transporter	-1.83	-1.42
SPBC1683.08	ght4	plasma membrane hexose:proton symporter	-1.81	-1.47
SPAC1002.17c	urg2	uracil phosphoribosyltransferase	-1.76	-1.55
SPBC1271.07c		N-acetyltransferase	-1.72	-1.62
SPAC977.15		dienelactone hydrolase family, implicated in cellular detoxification	-1.70	-1.47
SPBC359.05	abc3	vacuolar heme ABC transmembrane exporter Abc3	-1.61	-1.28
SPAC1002.18	urg3	DUF1688 family fungal conserved protein	-1.48	-1.24
SPAC1399.04c	uck2	uracil phosphoribosyltransferase Uck2	-1.48	-1.27
SPCC1884.01		Schizosaccharomyces pombe specific protein	-1.44	-1.87
SPAC13G7.12c	eki1	choline/ethanolamine kinase Eki1	-1.44	-1.22
SPBC32H8.02c	nep2	NEDD8 protease Nep2	-1.42	-1.16
SPCC1682.08c	mpf2	meiotic pumilio family RNA-binding protein Mpf2	-1.42	-1.29
SPBC1683.09c	frp1	plasma membrane ferric-chelate reductase Frp1	-1.41	-1.06
SPAC521.03		short chain dehydrogenase, human DHRS7 family	-1.40	-1.06
SPAC11D3.03c		aminomethyltransferase-like and DUF1989 family protein	-1.39	-1.77
SPBC1271.08c		Schizosaccharomyces pombe specific protein	-1.38	-1.01
SPCC584.16c		Schizosaccharomyces specific protein	-1.37	-1.22
SPAC10F6.07c	mug94	Schizosaccharomyces pombe specific protein Mug94	-1.35	-1.66
SPAC56E4.03		aromatic aminotransferase	-1.29	-1.00
SPCC794.03		amino acid transmembrane transporter	-1.29	-1.04
SPBC25B2.11	pof2	F-box protein Pof2	-1.29	-1.20
SPCC16A11.01	sfk1	plasma membrane protein involved in inositol lipid-mediated signaling	-1.19	-1.14
SPAC25B8.13c	isp7	2-OG-Fe(II) oxygenase superfamily protein	-1.17	-1.05
SPBC409.11	meu18	Schizosaccharomyces specific protein Meu18	-1.16	-1.06
SPCC569.07		aromatic aminotransferase	-1.16	-1.32
SPBC1773.17c	gor2	glyoxylate reductase	-1.09	-1.17
SPBPB21E7.08		pseudogene	-1.08	-1.15
SPCC965.14c		cytosine deaminase	-1.08	-1.27
SPBC1773.14	arg7	argininosuccinate lyase	-1.05	-1.02
SPAC11D3.02c		ELLA family acetyltransferase	-1.03	-1.11
