# Supplementary material for: Genetic suppressor screen identifies Tgp1 (glycerophosphocholine transporter), Kcs1 (IP6 kinase), and Plc1 (phospholipase C) as determinants of inositol pyrophosphate toxicosis in fission yeast
Source: mBio. 2023 Dec 22;15(2):e03062-23. doi: 10.1128/mbio.03062-23 (PMC10865970; doi:10.1128/mbio.03062-23)
Supplement: Table S2 — Genes that are dysregulated in kcs1 mutants. [file mbio.03062-23-s0003.pdf]

SystematicID	Gene.name	Product	R332T_log2FoldChange
SPBPB21E7.04c	cmt2	O-methyltransferase, human COMT catechol homolog 2	2.96
SPCC18B5.02c		cinnamoyl-CoA reductase pseudogene	2.24
SPAC11D3.01c		Con-6 family conserved fungal protein	2.07
SPBC4F6.09	str1	plasma membrane siderophore-iron transmembrane transporter Str1	2.06
SPBC106.02c	srx1	sulfiredoxin	1.98
SPCC1235.14	ght5	plasma membrane high-affinity glucose/fructose:proton symporter	1.75
SPAC750.01		NADP-dependent aldo/keto reductase	1.70
SPCC663.08c		short chain dehydrogenase, unknown specificity	1.67
SPAC22F3.02	atf31	DNA-binding transcription factor Atf31	1.50
SPAC1F7.07c	fip1	plasma membrane iron transmembrane transporter Fip1	1.38
SPBC1271.07c		N-acetyltransferase	1.35
SPAPB24D3.08c		NADP-dependent oxidoreductase	1.28
SPCC1020.09	gnr1	heterotrimeric G protein beta (WD repeat) subunit Gnr1	1.18
SPCC70.08c		methyltransferase	1.16
SPBPB10D8.02c		arylsulfatase	1.14
SPCC794.03		amino acid transmembrane transporter	1.14
SPCC965.07c	gst2	glutathione S-transferase Gst2	1.10
SPBC1683.09c	frp1	plasma membrane ferric-chelate reductase Frp1	1.07
SPAC13G7.02c	ssa1	Hsp70 family heat shock protein Ssa1	1.07
SPAC1F7.08	fio1	plasma membrane iron transport multicopper oxidase Fio1	1.02

SystematicID	Gene.name	Product	R332T_log2FoldChange
SPBP4G3.02	pho1	extracellular acid phosphatase Pho1	-6.00
SPBC8E4.01c	pho84	plasma membrane inorganic phosphate transmembrane transporter	-4.92
SPBPB2B2.06c		extracellular 5'-nucleotidase, human NT5E family	-4.72
SPBC8E4.12c	ecl3	extender of chronological lifespan protein Ecl3	-3.52
SPAC750.05c	ftm4	sub-telomeric 5Tm protein family Ftm4	-2.61
SPBC1271.09	tgp1	plasma membrane glycerophosphodiester transmembrane transporter	-2.35
SPBC29B5.02c	isp4	plasma membrane OPT oligopeptide transmembrane transporter family	-2.29
SPBPB2B2.01		amino acid transmembrane transporter	-1.71
SPAC343.12	rds1	ferritin related conserved fungal protein	-1.56
SPBC354.12	gpd3	glyceraldehyde 3-phosphate dehydrogenase Gpd3	-1.37
SPBC25B2.08		Schizosaccharomyces pombe specific protein	-1.12
SPBC1861.02	abp2	unknown protein, may bind replication origins Abp2	-1.09
SPAC2H10.01		DNA-binding transcription factor, zf-fungal binuclear cluster type	-1.09
SPBC36.03c	mfs3	plasma membrane spermidine transmembrane transporter Mfs3	-1.08
SPBC1683.01	pho841	plasma membrane inorganic phosphate transmembrane transporter Pho841	-1.08
SPBC16D10.06	zrt1	plasma membrane ZIP zinc transmembrane transporter Zrt1	-1.08
SPAC23D3.14c	aah2	alpha-amylase homolog Aah2	-1.05
SPBC947.04	pfl3	cell surface glycoprotein, flocculin Pfl3, DIPSY family	-1.00

SystematicID	Gene.name	Product	L338R_log2FoldChange
SPCC1235.14	ght5	plasma membrane high-affinity glucose/fructose:proton symporter	2.32
SPAC27D7.03c	mei2	RNA-binding protein involved in meiosis Mei2	1.75
SPAC15E1.02c		DUF1761 family protein	1.71
SPAC13G7.02c	ssa1	Hsp70 family heat shock protein Ssa1	1.63
SPAP8A3.04c	hsp9	heat shock protein Hsp9	1.42
SPAC637.03		DUF1774 family multi-spanning conserved fungal membrane protein	1.37
SPBC21C3.19	rtc3	SBDS family protein Rtc3	1.32
SPCC70.08c		methyltransferase	1.29
SPBC106.02c	srx1	sulfiredoxin	1.25
SPCC548.07c	ght1	plasma membrane high-affinity glucose:proton symporter	1.20
SPBC16E9.16c	lsd90	Lsd90 protein	1.13
SPAC11D3.19		Schizosaccharomyces pombe specific protein	1.12
SPCC338.12	pbi2	vaculoar proteinase B inhibitor Pbi2	1.10
SPAC21E11.04	aca1	L-azetidine-2-carboxylic acid acetyltransferase Aca1	1.07
SPAC1F7.07c	fip1	plasma membrane iron transmembrane transporter Fip1	1.02
SPBC725.10	tps0	mitochondrial outer membrane protein	1.01

SystematicID	Gene.name	Product	L338R_log2FoldChange
SPAC750.05c	ftm4	sub-telomeric 5Tm protein family Ftm4	-8.15
SPAC186.05c	gdt1	Golgi calcium and manganese antiporter Gdt1	-5.70
SPAC750.01		NADP-dependent aldo/keto reductase	-5.14
SPBC8E4.12c	ecl3	extender of chronological lifespan protein Ecl3	-2.17
SPBPB2B2.06c		extracellular 5'-nucleotidase, human NT5E family	-1.99
SPBP4G3.02	pho1	extracellular acid phosphatase Pho1	-1.77
SPBC1271.09	tgp1	plasma membrane glycerophosphodiester transmembrane transporter	-1.62
SPCC364.06	nap1	histone H2A-H2B chaperone Nap1	-1.48
SPBC8E4.01c	pho84	plasma membrane inorganic phosphate transmembrane transporter	-1.43
SPBC947.04	pfl3	cell surface glycoprotein, flocculin Pfl3, DIPSY family	-1.31
SPAC1039.02		extracellular 5'-nucleotidase, human NT5E family	-1.31
SPAC1002.19	urg1	GTP cyclohydrolase II Urg1	-1.27
SPBC25B2.08		Schizosaccharomyces pombe specific protein	-1.26
SPBPB10D8.03		pseudogene transporter	-1.24
SPBC29B5.02c	isp4	plasma membrane OPT oligopeptide transmembrane transporter family	-1.23
SPBC36.03c	mfs3	plasma membrane spermidine transmembrane transporter Mfs3	-1.23
SPBC1861.01c	cnp3	CENP-C ortholog Cnp3	-1.14
SPAC1002.17c	urg2	uracil phosphoribosyltransferase	-1.11
SPBC3H7.05c		mitochondrial Membrane Bound O-Acyl Transferase (MBOAT) family	-1.09
SPAC23D3.05c		alcohol dehydrogenase pseudogene	-1.08
SPBPB7E8.01		Schizosaccharomyces specific protein, predicted GPI anchor	-1.04
SPAC17C9.16c	mfs1	plasma membrane spermidine transmembrane transporter Mfs1	-1.03
SPAC11D3.05	mfs2	transmembrane transporter Mfs2	-1.01

SystematicID	Gene.name	Product	E834K_log2FoldChange
SPCC1235.14	ght5	plasma membrane high-affinity glucose/fructose:proton symporter	2.45
SPAC15E1.02c		DUF1761 family protein	1.68
SPAC27D7.03c	mei2	RNA-binding protein involved in meiosis Mei2	1.65
SPCC70.08c		methyltransferase	1.40
SPAC13G7.02c	ssa1	Hsp70 family heat shock protein Ssa1	1.36
SPAP8A3.04c	hsp9	heat shock protein Hsp9	1.29
SPAC637.03		DUF1774 family multi-spanning conserved fungal membrane protein	1.26
SPBC106.02c	srx1	sulfiredoxin	1.21
SPAC11D3.19		Schizosaccharomyces pombe specific protein	1.21
SPAC1F7.07c	fip1	plasma membrane iron transmembrane transporter Fip1	1.18
SPAC11D3.01c		Con-6 family conserved fungal protein	1.18
SPCC548.07c	ght1	plasma membrane high-affinity glucose:proton symporter Ght1	1.15
SPAC21E11.04	aca1	L-azetidine-2-carboxylic acid acetyltransferase Aca1	1.14
SPBPB2B2.12c	gal10	UDP-glucose 4-epimerase/aldose 1-epimerase Gal10	1.14
SPCC663.08c		short chain dehydrogenase, unknown specificity	1.14
SPAC186.01	pfl9	cell surface glycoprotein, flocculin Pfl9, DIPSY family	1.11
SPBC1271.07c		N-acetyltransferase	1.07
SPAC1039.09	isp5	amino acid transmembrane transporter Isp5	1.03
SPCC338.12	pbi2	vaculoar proteinase B inhibitor Pbi2	1.03
SPBC16E9.16c	lsd90	Lsd90 protein	1.03
SPBC21C3.19	rtc3	SBDS family protein Rtc3	1.00

SystematicID	Gene.name	Product	E834K_log2FoldChange
SPAC750.05c	ftm4	sub-telomeric 5Tm protein family Ftm4	-6.74
SPAC750.01		NADP-dependent aldo/keto reductase	-4.53
SPAC186.05c	gdt1	Golgi calcium and manganese antiporter Gdt1	-4.15
SPBPB2B2.06c		extracellular 5'-nucleotidase, human NT5E family	-2.17
SPBC8E4.12c	ecl3	extender of chronological lifespan protein Ecl3	-2.02
SPBP4G3.02	pho1	extracellular acid phosphatase Pho1	-1.95
SPBC1271.09	tgp1	plasma membrane glycerophosphodiester transmembrane transporter	-1.60
SPBC29B5.02c	isp4	plasma membrane OPT oligopeptide transmembrane transporter family	-1.56
SPBC8E4.01c	pho84	plasma membrane inorganic phosphate transmembrane transporter	-1.47
SPCC364.06	nap1	histone H2A-H2B chaperone Nap1	-1.42
SPBC36.03c	mfs3	plasma membrane spermidine transmembrane transporter Mfs3	-1.41
SPAC1002.19	urg1	GTP cyclohydrolase II Urg1	-1.35
SPBC947.04	pfl3	cell surface glycoprotein, flocculin Pfl3, DIPSY family	-1.28
SPBC25B2.08		Schizosaccharomyces pombe specific protein	-1.28
SPBPB7E8.01		Schizosaccharomyces specific protein, predicted GPI anchor	-1.22
SPBC3H7.05c		mitochondrial Membrane Bound O-Acyl Transferase (MBOAT) family	-1.17
SPAC1002.17c	urg2	uracil phosphoribosyltransferase	-1.17
SPAC1039.02		extracellular 5'-nucleotidase, human NT5E family	-1.15
SPAC17C9.16c	mfs1	plasma membrane spermidine transmembrane transporter Mfs1	-1.11
SPBC26H8.11c	the4	acyl-coenzyme A thioesterase The4	-1.11
SPBPB10D8.03		pseudogene transporter	-1.10
SPAC13G7.13c	msa1	RNA-binding protein Msa1	-1.09
SPBPB21E7.08		pseudogene	-1.09

SystematicID	Gene.name	Product	R332T_log2FoldChange	L338R_log2FoldChange	E834K_log2FoldChange
SPCC1235.14	ght5	plasma membrane high-affinity glucose/fructose:proton symporter	1.75	2.32	2.45
SPCC70.08c		methyltransferase	1.16	1.29	1.40
SPAC13G7.02c	ssa1	Hsp70 family heat shock protein Ssa1	1.07	1.63	1.36
SPBC106.02c	srx1	sulfiredoxin	1.98	1.25	1.21
SPAC1F7.07c	fip1	plasma membrane iron transmembrane transporter Fip1	1.38	1.02	1.18

SystematicID	Gene.name	Product	R332T_log2FoldChange	L338R_log2FoldChange	E834K_log2FoldChange
SPAC750.05c	ftm4	sub-telomeric 5Tm protein family Ftm4	-2.61	-8.15	-6.74
SPBC8E4.12c	ecl3	extender of chronological lifespan protein Ecl3	-3.52	-2.17	-2.02
SPBPB2B2.06c		extracellular 5'-nucleotidase, human NT5E family	-4.72	-1.99	-2.17
SPBP4G3.02	pho1	extracellular acid phosphatase Pho1	-6.00	-1.77	-1.95
SPBC1271.09	tgp1	plasma membrane glycerophosphodiester transmembrane transporter	-2.35	-1.62	-1.60
SPBC8E4.01c	pho84	plasma membrane inorganic phosphate transmembrane transporter	-4.92	-1.43	-1.47
SPBC947.04	pfl3	cell surface glycoprotein, flocculin Pfl3, DIPSY family	-1.00	-1.31	-1.28
SPBC25B2.08		Schizosaccharomyces pombe specific protein	-1.12	-1.26	-1.28
SPBC29B5.02c	isp4	plasma membrane OPT oligopeptide transmembrane transporter family	-2.29	-1.23	-1.56
SPBC36.03c	mfs3	plasma membrane spermidine transmembrane transporter Mfs3	-1.08	-1.23	-1.41
